# Persistent type I interferon signaling within the brain of people with HIV on ART with cognitive impairment

**DOI:** 10.1371/journal.ppat.1013411

**Published:** 2025-08-20

**Authors:** Yuyang Tang, Ling Xie, Ciniso Sylvester Shabangu, Dajiang Li, Gabriela da Silva Prates, Ashokkumar Manickam, Lilly M. Wong, Antoine Chaillon, Edward P. Browne, Sara Gianella, Wenzhe Ho, David M. Margolis, Xian Chen, Wenhui Hu, Guochun Jiang

**Affiliations:** 1 Department of Medicine and UNC HIV Cure Center, University of North Carolina at Chapel Hill, Chapel Hill, North Carolina, United States of America; 2 Department of Biochemistry and Biophysics, University of North Carolina at Chapel Hill, Chapel Hill, North Carolina, United States of America; 3 Department of Medicine, University of California, San Diego, La Jolla, California, United States of America; 4 Department of Pathology and Laboratory Medicine, Temple University Lewis Katz School of Medicine, Philadelphia, Pennsylvania, United States of America; 5 Department of Anatomy and Neurobiology, Virginia Commonwealth University, Richmond, Virginia, United States of America; NIH, NIAID, UNITED STATES OF AMERICA

## Abstract

To better understand the molecular mechanism that drives neuroinflammation, we analyzed the protein profiles of 27 brains from HIV with HIV (PWH) on antiretroviral therapy (ART), including various stages of HIV-associated neurocognitive disorders (HAND), and compared them to 9 HAND-negative controls. We found that most of the proteins that were increased—about 66.7%—were involved in immune response pathways. Of these, 23.3% were specifically related to type I interferon (IFN-I) signaling, which remains active in the brain through both HIV-related and unrelated mechanisms. Using single-cell RNA sequencing (scRNA-seq) on brain tissues collected during rapid autopsies from participants in the Last Gift cohort, we found that IFN-I signaling was especially strong in astrocytes, microglia (MG), and endothelial cells. In a mini-brain organoid model of acute HIV infection, IFN-I signaling was also highly active in astrocytes but less so in MG. Interestingly, IFN-I activation can happen without HIV being present—expression of human endogenous retrovirus-W1 (HERV-W1) Env can directly trigger this response in astrocytes, and it continues in glial cells even with effective ART. Together, our findings point to persistent IFN-I activation in glial and endothelial cells in the brain, which may contribute to neuroinflammation and cognitive disorders in PWH on ART.

## Author summary

Up to 50% of people with HIV (PWH) experience HIV-associated neurocognitive disorders (HAND), even in the era of modern antiretroviral therapy (ART). However, the underlying molecular mechanisms of HAND pathogenesis remain poorly understood. By analyzing protein profiles from 27 HIV-positive brains at various stages of HAND and comparing them to 9 HAND-negative controls, we found that 73.3% of elevated proteins were associated with immune response pathways, particularly type I interferon (IFN-I) signaling. Single-cell RNA sequencing revealed strong IFN-I activity in astrocytes, microglia, and endothelial cells. Mini-brain organoid models confirmed heightened IFN-I activation in astrocytes during acute HIV infection. Notably, IFN-I signaling was also triggered by HERV-W1 Env expression, independent of HIV presence. These findings suggest that persistent IFN-I activation may contribute to chronic neuroinflammation and cognitive impairment in people with HIV, even under effective modern ART.

## Introduction

Effective antiretroviral therapy (ART) has lessened the severity of HIV-associated neurocognitive diseases (HAND). However, it has failed to reduce the overall frequency of neurocognitive impairment [1,2]. Therapeutic approaches focusing only on improving or intensifying ART regimens are insufficient to decrease HAND occurrences. Therefore, there is an urgent need to dissect the major players that contribute to cognitive disorders in the era of modern ART.

In the pre-ART era, HAND was associated with persistent neuroinflammation that has increased microglial activation, aberrant expression of inflammatory cytokines, and reactive oxygen species. As a result, neuroinflammation is associated with neurodegeneration and brain damage in HAND [1,2]. However, the underlying mechanisms of neuroinflammation remain unclear. During the long-term ART, a low level of immune activation persists in the brain, evidenced by macrophage/microglia activation and increased immune activation in people with HIV (PWH) [3–5]. This has raised the question of how immune activation is maintained in the brain, especially during effective ART, in which HIV-induced innate immune responses should have been diminished. Finally, it remains underdetermined whether persistent neuroinflammation plays an essential role in neuronal damage for HAND pathogenesis.

To address these questions, we need to detail the players that drive the brain immune activation in PWH with HAND. In this study, we aim to advance the immune activation status and to elucidate the drivers of persistent neuroinflammation by quantitative proteomic analysis using HAND brains on ART and single-cell RNA-seq using the brain tissues in PWH from the Last Gift Program. Our findings point to persistent type I interferon (IFN-I) signaling as one of the major drivers of neuroinflammation in the brain, which may orchestrate with human endogenous retrovirus W1 (HERV-W1) Env pathogenic activation as an additional root of the chronic immune activation in the brain of PWH with HAND even under effective ART.

## Results

### Characteristics of HAND cohort

Brain tissues from 27 PWH and 9 people without HIV were included in this study. Participants were enrolled as part of the National NeuroAIDS Tissue Consortium (NNTC) between 1999 and 2009 (S1 Table), which has established a unique brain bank with high-quality and well-characterized specimens from PWH that have comprehensive neuromedical, neuropsychological, and psychiatric data before death. Demographics, clinical characteristics, and laboratory data were summarized in S1–S3 Tables. Most participants were male (85.2%) with a median age of 44 years. 69.4% were white, and 8.3% were black. The median duration of HIV infection was 8 years. PWH were further divided into groups with (n = 19) or without (n = 8) HAND. Among the neurocognitive impaired individuals, 10 had HIV-associated dementia (HAD) while 9 had a mild neurocognitive disease (MND) or asymptomatic neurocognitive impairment (ANI). All participants were on ART, whereas most PWH had detectable viral loads near or at the time of death (70.3% with plasma viral loads >400 copies/mL). This may be due to the poor ART adherence in some of these PWH, particularly at the end of days before death, which may be related to the relatively high incidence of HAND. This provided us with a unique opportunity to study the mechanism of HAND in the era of ART. Lastly, we included the analyses of rapid autopsy brain samples from altruistic PWH on suppressive ART (S4 Table) who were enrolled in the “Last Gift” Program and the National Disease Research Interchange (NDRI) [6,7]. These samples allowed us to perform single-cell (sc) analysis to further define neuroinflammation in brains from PWH receiving suppressive ART. Because multiple cohorts were analyzed in this study, we referred to these individuals as “PWH on ART” or, in the case of brain samples, as “HAND brain on ART”.

### IFN-I activation signature in the brains of PWH with HAND

We performed tandem mass tag (TMT) quantitative proteomic analyses using the snap-frozen brain tissues from PWH with HAND on ART (n = 19), comparing them with the brains of PWH without cognitive impairment (n = 8) and controls without HIV infection (n = 9). From each PWH, we collected frozen brain tissues from the deep white matter (WM) regions (frontal lobe) (S2, S3 Tables). WM sections from 7 people without HIV were included as controls (S5 Table). We have also included frontal lobe gray matter (GM) from a subset of 8 PWH with HAND (6 cases with HAD, 2 cases with MND, S2 Table), a subset of 3 cases of PWH without HAND, and 2 controls without HIV (S2, S3 Tables).

**Fig 1:**
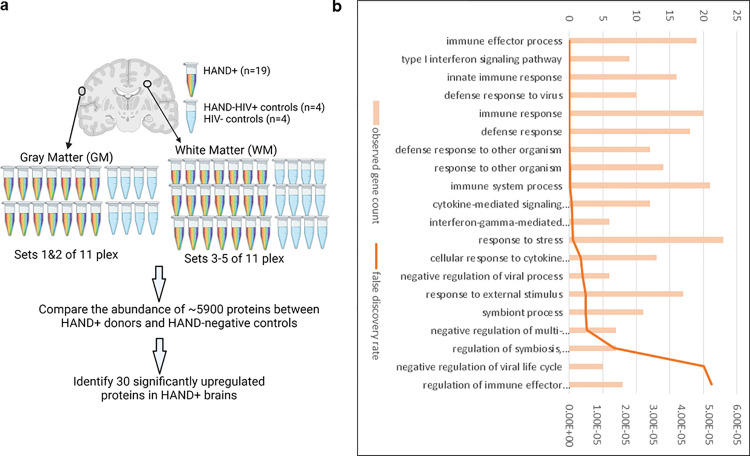
Quantitative proteomic analysis to define the protein signature in the HAND brains. A Proteomic Experimental Design: Five sets of 11-plex Tandem Mass Tags (TMT) were used to quantify and identify proteins in HAND+ brain samples compared to control brain samples. The figure was created using BioRender. B The top biological pathways identified from the 30 significantly altered proteins in HAND+ brains.

Since TMT quantitative proteomics enables multiplexing of up to 11 samples in each run [8], the tissue samples were divided into several sets (sets 1&2 for GM and sets 3–5 for WM), each of which contained 5–7 samples from PWH with HAND and 4–6 controls (Fig 1A). This design reduced the batch effects and allowed us to maximally quantify the subtle changes in the abundance of each protein. A difference in abundance of 5905 brain proteins was detected and analyzed. Among them, 30 proteins were significantly upregulated in HAND brains (S6 Table). These proteins belonged to the activation of >20 biological pathways, most of which were related to the innate immune response (Fig 1B, S7 Table).

**Fig 2:**
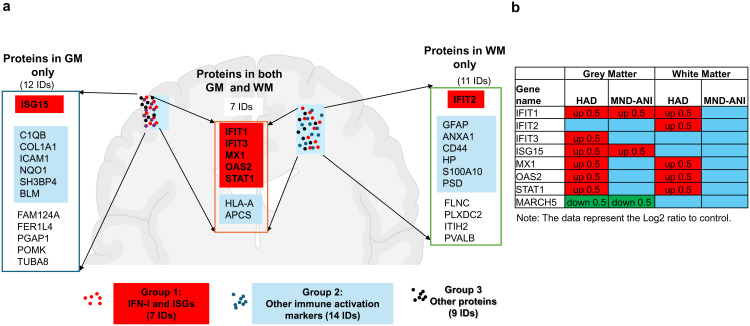
Analysis of the proteins identified in the TMT proteomic profiles in the HAND brains. A Category of the proteins and their localization in HAND brains. The figure was created using BioRender. B Up-regulated protein list related to the activation of IFN-I signaling in the HAND brains.

The 30 upregulated proteins can be categorized into 3 groups based on pathway analyses: Group 1 included ISGs from the IFN-I pathway; Group 2 included other immune activation markers; and Group 3 was composed of non-immune factors. About 23% (7 proteins) of these upregulated proteins were within the IFN-I pathway, including STAT1, IFIT1, IFIT2, IFIT3, ISG15, MX1, and OAS2 (Fig 2A, 2B). Nineteen upregulated proteins were found within the GM region while 18 proteins were found in the WM region, including 7 upregulated proteins (5 ISGs or 2 other immune markers) found in both brain regions (Fig 2A). The upregulation of IFN-I pathway proteins (n = 5, IFIT1, IFIT3, MX1, OAS2, and STAT1) was observed in both brain sub-regions (Fig 2). While the majority of ISG protein upregulation was observed in the HAD brain, increased IFIT1 and ISG15 proteins in GM were detected in the MND/ANI brain (Fig 2B), suggesting that IFN-I activation has a role in the early stages of HAND pathogenesis. The differential IFN-I activation between the GM and WM regions (Fig 2 and S6 Table) may reflect a region-specific, persistent IFN-I activation response. Interestingly, we found that the negative regulator of the IFN-I signaling, MARCH5 [9], was downregulated in the GM from both PWH with HAD and MND/ANI (Fig 2B and S7 Table). This may further reinforce the overall IFN-I activation to promote HAND. Together, these data point to a persistent activation of IFN-I signaling within brain regions in PWH with HAND.

**Fig 3:**
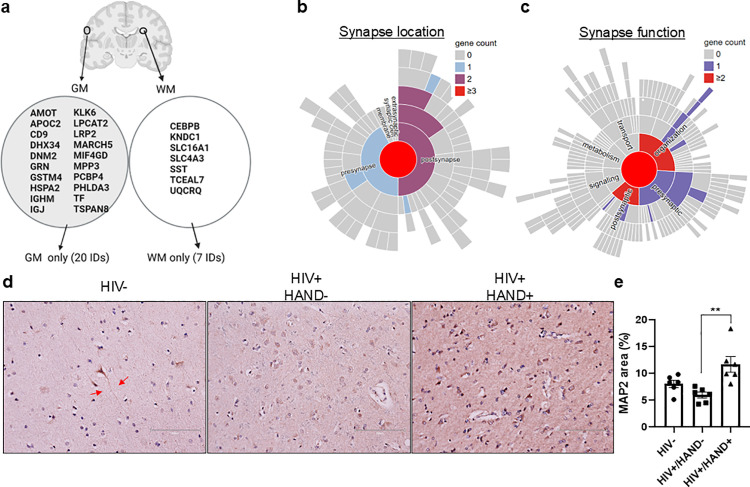
Analysis of the down-regulated proteins identified in the TMT proteomic profiles in the HAND brains. A A category of down-regulated proteins in the HAND brains. B-C SynGo analysis of down-regulated proteins in synapse structure and function. The gene count column indicates the quantity of distinctive dysregulated genes annotated in SynGO related to this term or its subcategories. D IHC of MAP2 showed synapse dysfunction in the HAND brains. Arrow, axons. E Quantitation of MAP2 after IHC in the HAND brains (n = 6). ***, *p* < 0.001; **, *p* < 0.01, by two-way ANOVA.

TMT proteomics also identified 27 down-regulated proteins (S8 Table). However, no significant pathways were identified by an initial IPA analysis. These proteins were highly expressed in the CNS (Fig 3A and S9 Table) with important neuronal functions. On top of that, AMOT is a key player in neuronal differentiation. Defective AMOT protein causes synapse developmental defects [10], leading to CNS disorders such as autism. KNDC1, also called v-KIND, is expressed in cerebellar granule cells, which are essential for normal motor coordination and balance [11]. MPP3 maintains an apical junctional complex and neuronal migration. MPP3 dysfunction delays the migration of progenitor cells during cortex development [12]. Lastly, TSPAN8, also called CD81, is a critical regulator of neuron-induced astrocyte proliferative regulation [13]. With SynGo, an evidence-based tool specialized for synapse function [14], we found that many of these proteins are localized in the synapse, and dysregulation of these proteins may impact the defects in synapse and pre (or post) synapse function (Fig 3B–3C and S10 Table). These proteins were associated with myeloid cell iron uptake in neurodegenerative disorders [15], Alzheimer’s disease (by the impairment of amyloid β traffic and degradation), frontotemporal dementia, and defects in endosomal recycling [16] (S11 Table). Immunostaining analyses showed that MAP2 protein was enriched in the cell body in HIV + /HAND+ compared with HIV+ HAND- brains, resulting in synapse reduction (Fig 3D–3E). This is in line with a previous report, where PWH who had HAND had greater MAP2 concentrations within the CSF than cognitive normal PWH [17]. These data support that the dysfunction of these brain proteins is associated with synapse dysfunction related to HAND pathogenesis.

### Aberrant brain IFN-I activation and HAND pathogenesis in PWH

**Fig 4:**
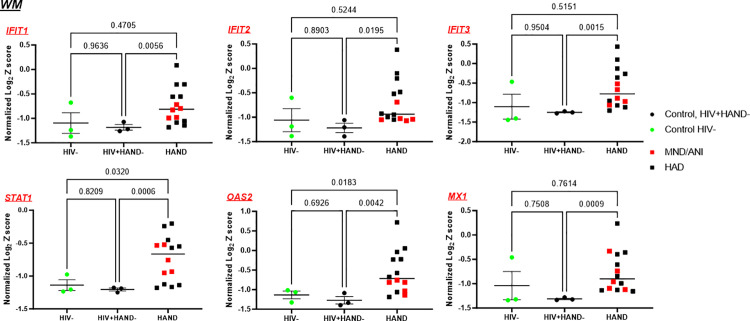
IFN-I signaling proteins are activated in HAND brains. IFN-I signaling proteins were up-regulated in the WM of HAND brains. The normalized Z-scores of the proteins in HAND brains (n = 14) were compared with that of the HIV- control brain samples (n = 3) or to HIV+HAND- brains (n = 3). The p-value was calculated with the Brown-Forsythe Welch ANOVA multiple comparison test.

The data within the same TMT set enabled us to accurately measure the subtle abundance changes of related proteins. Next, we analyzed further the changes in proteomic signatures across all 5 sets of brain samples. The abundance scores (Z scores) of each brain sample were normalized to control Z scores in the same set, which was calculated as below: [Normalized Z score = Z score of each sample/the average of Z scores of controls in the same set-1]. We then compared the normalized Z scores between PWH with HAND and controls (i.e., PWH without HAND and controls without HIV). In the WM regions, we compared 14 PWH with HAND (9 with HAD, 5 with MND/ANI) with 6 controls (3 without HIV and 3 PWH without HAND). As shown in Fig 4, this combinational analysis further confirmed the significant upregulation of IFN-I proteins (IFIT1, IFIT2, IFIT3, STAT1, OAS2, and MX1) in the WM regions of HAND compared with PWH without HAND (HIV+HAND-). The significant upregulation of STAT1 and OAS2 was also detected when compared with HIV-negative (HIVneg) controls. No significant difference was observed between the HIVneg group and HIV+ HAND- group. In the GM region, there were 11 brain samples from PWH with HAND (7 with HAD, 4 with MND/ANI) and 4 control brain samples (2 without HIV and 2 PWH without HAND). Considering that we only had two control samples from each control group, in which they showed comparable levels of the analyzed proteins, the data from the HAND groups were compared with the combined control groups. As shown in S1 Fig, the abundance of IFIT1, IFIT3, MX1, ISG15, and STAT1 was significantly higher in the GM of brains from PWH with HAND. Notably, ISG15 was only enriched in the GM region of brains in PWH with HAND, but not in the WM (Figs 2 and S1, S6 Table). Elevated IFIT2 and OAS2 were also detected in GM of HAND brains but were not significant during the combinational analysis (S1 Fig). Taken together, a persistent activation of IFN-I signaling signature exists in the HAND brains from PWH.

### Other immune activation markers and non-immune proteins in the HAND brains

**Fig 5:**
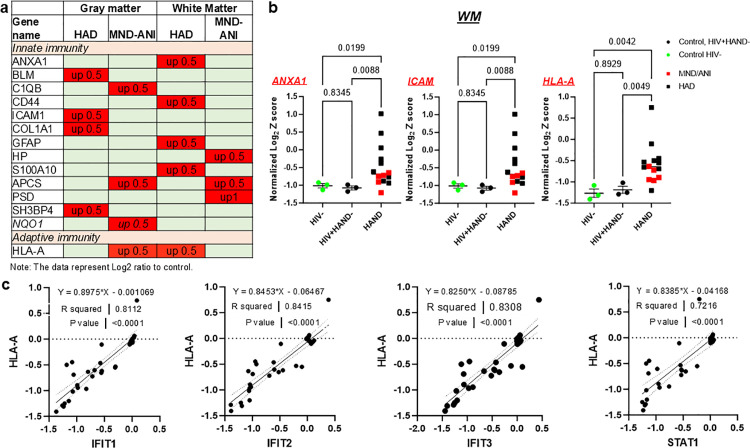
Immune activation in HAND brains. A Immune activation markers identified by TMT proteomic assays in HAND brains. B The normalized Z-score of the proteins in WM of HAND brains (n = 14) was compared with that of the HIV- control brain samples (n = 3) or HIV+HAND- brains (n = 3). The p-value was calculated with the Brown-Forsythe Welch ANOVA multiple comparison test. C The abundance of HLA-A was positively correlated with the protein level of IFIT1, IFIT2, IFIT3, and STAT1. Data were analyzed using linear regression.

In addition to the IFN-I activation, we observed an upregulation of 13 other innate immune-related proteins, including ANXA1, ICAM1, COL1A1, and C1QB as well as glia activation marker proteins, CD44, and GFAP (Fig 5A). Combinational analysis across different sets of samples confirmed the significant upregulation of ANXA1, ICAM in the WM (Fig 5B), and C1QB and NQO1 in the GM of brains from PWH with HAND (S2 Fig). Interestingly, the adaptive immune response marker HLA-A was upregulated in the brains of PWH (HAD WM set and MND/ANI GM set) (Fig 5A). Combinational analysis of HLA-A expression further confirmed a significant upregulation in WM (Fig 5B) but not in GM of HAND brains (S2 Fig), consistent with a previous brain transcriptomic report [18]. The abundance of HLA-A was positively associated with IFIT1, IFIT2, IFIT3, and STAT1 expression (Fig 5C), whereas ICAM expression was positively correlated with STAT1 and IFIT3 (S3A, S3B Fig). IFN-I activation is known as an ambiguous modulator to promote innate immunity and subsequently shape the adaptive immune response. These observations imply that there exists both innate and adaptive immune activation in the HAND brains, which may interplay with each other after IFN-I activation within the brains of PWH with HAND. Of note, the ISG protein IFIT1 was negatively associated with KLK6 [19], CD9 [20], and TSPAN8 [21] (S3C Fig), well-known proteins essential for neuronal functioning [13,22,23]. We also detected 8 non-immune related proteins upregulated in the brains, such as the metabolism-related markers (S12 Table). These proteins were only upregulated in HAD brains on ART, which may not be related to HAND on modern ART, i.e., ANI and MND forms of HAND. Together, PWH with HAND have a persistent immune activation in their brains. Given the established role of IFN-I signaling in shaping the innate and adaptive immune response and the strong association between IFN-I activation and other innate/adaptive immune proteins [24–26], our data suggest that the IFN-I pathway may be at the center of driving persistent immune activation in the brains of PWH with HAND on ART, leading to neuronal impairments (Figs 3D and S3C).

### Validation of sustained IFN-I activation and persistent immune activation in brains of PWH with HAND

**Fig 6:**
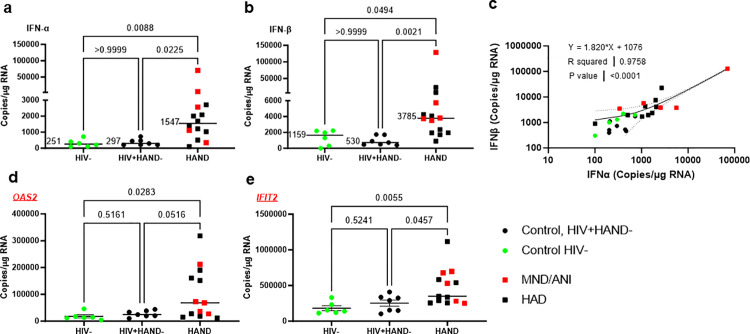
Expression of IFNα and IFNβ in HAND brains. IFNα (A), IFNβ (B), OAS2 (D), and IFIT2 (E) mRNA expression was analyzed by ddRT-qPCR in the WM of HAND brains (n = 14), compared with WM of HIV- control brain samples (n = 6) or HIV+HAND- brains (n = 7). The p-value was calculated by a Krukal-Wallis Test in comparison with controls. The mean values of IFNα and IFNβ were shown on the left. C The expression of IFNα and IFNβ was positively correlated. Data were analyzed using linear regression.

Next, we validated the IFN-I signature in brain tissues. Since the induction of ISG genes is mainly controlled by transcriptional regulation [27], we quantified the transcripts of IFNα/β and ISG genes as a surrogate for IFN-I activation *via* ddPCR. The WM from the same 9 HAD brains and 5 brains with MND/ANI were analyzed. In addition to the 6 controls used in the proteomic analysis, we added another 7 control brain samples (13 controls in total, 7 neurocognitive negative PWH, and 6 HIV uninfected controls). We observed significantly elevated levels of both IFNα and IFNβ transcripts in HAND brains (Fig 6A, 6B). The median levels of IFNα and IFNβ increased about 3–7-fold in the HAND brains, compared with the levels of IFNs detected in control brains (HIVneg donors and neurocognitive negative PWH). Notably, the median level of IFNβ was nearly 2-fold of the median level of IFNα in HAND brains (3,785 copies/µg RNA vs 1,547 copies/µg RNA) (Fig 6A, 6B). Also, we observed a strong correlation between the levels of IFNα and IFNβ transcripts (Fig 6C). We next measured the levels of ISG transcripts, the essential downstream genes after IFN-I signaling activation. Like TMT proteomics analysis, HAND brains had significantly higher levels of ISG transcripts, including OAS2 and IFIT2 (Fig 6D, 6E). The elevated levels of MX1, STAT1, and HLA-A were also detectable in HAND brains but did not reach significant levels compared with neurocognitive or uninfected controls (S4 Fig), reflecting a possible discrepancy of RNA *vs* protein expression of downstream IFN-I signaling. Together, these results largely validated sustained IFN-I activation in HAND brains on ART.

### IFN-I signaling in the subsets of CNS cells in the brains of PWH on suppressive ART and our microglia-containing cerebral organoid (MCO) model during acute HIV infection

**Fig 7:**
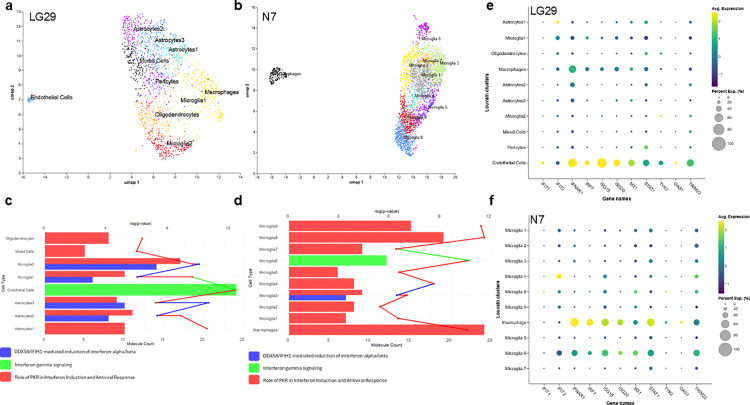
Single-cell RNA-seq analysis of IFN-I signaling in the CNS cells and brain tissues from PWH on ART. CNS single-cell suspension (LG29, A) or purified MG (N7, B) was subjected to scRNA-seq analyses to characterize CNS cells, cell subsets, and MG clusters. IFN-I signaling activation in the MG of brains from PWH LG29 (ART-suppressed, C and E) and N7 (shortly ART-interrupted, D and F). The size of each circle represents the percentage of cells in the cluster where the gene is detected.

Our data revealed IFN-I activation in HAND brains on ART. The presence of many cell types within the brain, such as microglia, astrocytes, neurons, oligodendrocytes, and possibly T cells, makes it challenging to identify which CNS cell subset (s) drives IFN-I activation. One powerful tool used to address this important question is single-cell RNA-seq (scRNA-seq) [28]. We then utilized fresh human brain tissues from 2 PWH under durable ART (LG29 did not stop ART and remained viral suppression until death, and N7 stopped ART 7 days before death) (S4 Table). The brain tissues were collected *via* the established rapid research autopsy procedures from ART-suppressed PWH enrolled in the “Last Gift” Program [7] and enrolled at NDRI [29]. The CNS cells were isolated using a recently validated protocol in our laboratory [6]. After isolating the CNS cells from fresh human brains, scRNA-seq was carried out on the bulk CNS cell suspension (LG29, Fig 7A) and purified microglia (MG) [6] (N7, Fig 7B). As expected, many glial cells were detected by scRNA-seq in LG bulky CNS cells, including microglia (MG), astrocytes, pericytes, and oligodendrocytes. Endothelial cells were also detectable, which were distinct from glial cell populations (Fig 7A). However, T cells were undetectable, which was consistent with our previous study showing that CNS T cells were extremely low [6]. Neurons were not detected, which may be largely due to that our CNS cell protocol contains a mechanical dissociation step, damaging neurons during the preparation of CNS single cells. Of note, we were able to detect at least two MG subsets and one macrophage cell population. Similarly, three astrocyte subset cells were identified. As we showed previously [6], isolated myeloid cells from N7 were highly pure (Fig 7B). Among most of these cells were brain MG, with a minor fraction of macrophages. Since these were purified MG, we were able to identify at least 9 MG subset cells (Figs 7B, S6, and S7), supporting the highly heterogeneous nature of brain MG [28].

Surprisingly, among brain myeloid cells, even though macrophages were identified in ART-suppressed PWH (LG29), IFN-I signaling activation was not detected in the Top 50 pathways. Instead, it was largely enriched in the brain myeloid cells. Additionally, IFN-I was also detected in other glial cells, including astrocytes and oligodendrocytes. The enrichment of IFN-I in endothelial cells suggested a blood-brain barrier injury. Unlike ART-suppressed PWH, in PWH with short-term ART-interruption (N7), IFN-I signaling was detected in both macrophages and brain MG, whereas 8 in 9 subsets of MG expressed IFN-I, except for subset MG6, which expressed IFNγ (Fig 7C–7F). Of note, under ART suppression, IFN-I signaling activation was enriched in endothelial cells. Together, these data support ongoing neuroinflammation in the brains of PWH, even on suppressive ART (Fig 7E–7F). Since many subset cells were identified in these glial cells, we also applied cell trajectory analysis based on their gene expression profiles to order cells along a putative lineage tree, which can help understand dynamic biological processes to identify potential biomarkers. Trajectory analysis showed a possible CNS cell differentiation route from astrocytes, pericytes, and oligodendrocytes to MG (S6C Fig). Within MG, MG subset 9 may be differentiated from MG subset 3, MG subset 1, to MG subset 5 while MG subset 2, subset 4, subset 6, subset 7, and subset 8 cells are distinct from other MG subsets (S7C Fig).

Due to the extremely low levels of HIV RNA expression in the brain during ART, we were unable to localize HIV transcripts within CNS cells, despite their detectability by ddPCR or RNAscope. However, because MG and possibly astrocytes [30], are the known major cells that harbor HIV proviruses in the brain [6], these observations suggest that residual viral RNA expression in the glial cells maintains IFN-I activation despite suppressive ART. Together, these data support neuroinflammation of IFN-I activation in the HAND brain on ART.

Cell death, apoptosis, and cell survival signaling pathways were altered in the CNS cells on ART. Many cell death and apoptosis pathways were down-regulated in MG and astrocytes, while cell survival signaling was upregulated in both PWH on ART (S8A, S8B Fig), suggesting a survival signaling activation occurred in these glial cells, which may be critical for maintaining the stable viral reservoirs *in vivo*. Notably, macrophages and some subsets of MG acquired survival advantages over the others. Thus, some MG subsets may represent the true viral reservoirs on persistent ART, consistent with our previous observations showing that isolated MG maintained their viability and slowly grew for >3 months *ex vivo* [29]. Nevertheless, we wanted to point out that while apoptosis and cell survival signaling could be altered post-viral infection, they may be impacted by our tissue processing, where neurons were lost. Future studies will be pursued to better understand the underlying mechanisms.

Lastly, emerging data showed upregulation of senescence in microglia and macrophages causes demyelination, leading to a reduction in remyelination [31]. Under ART suppression, significant senescence activation was observed in MG2 and endothelial cells, but less in other glial cells (S8C Fig). After ART interruption, significant senescence was activated in MG1, 2, 3, 7, 8, 9, as well as macrophages (S8C Fig). A similar pattern was observed in senescence-associated secretory phenotype (SASP) signaling activation among CNS cells on ART. Together, these data showed a complex scenario of CNS cell dysfunctional phenotypes upon HIV infection in the CNS on ART, which may contribute to HAND pathogenesis.

To further re-capture the IFN-I signaling signature during HIV infection, we explored a previously reported MG-containing organoid (MCO) model established in our lab by scRNA-seq [32]. It contains most of the CNS cells, including MG and astrocytes, and can be effectively infected by HIV [33]. Many CNS cells were also identified in our MCO model, including neural stem cells (NSC), neural progenitor cells, oligodendrocyte precursor cells (OPC), neurons, astrocytes, microglia, oligodendrocytes and neuronal cells (S9A Fig). Interestingly, unlike the chronic viral infection (Fig 7E), after acute HIV infection [33] IFN-I activation was primarily enriched in astrocytes and, to a lesser extent, in MG and NSC (S9B Fig). IFN-I was also expressed in neurons, where IFIT3, ISG15, and STAT3 were enriched, indicating a possible neuronal injury after acute HIV infection in the brain. Of note, OPCs were highly enriched by the expression of IFN-I signaling genes, including IFIT3, ISG15, JAK1, and STAT3 (S9B Fig). Different from IFN-I activation, HIV transcripts were mostly enriched in MG cells and possibly in OPCs, oligodendrocytes, and NSCs (S9C Fig). Since MG is the major target of HIV infection in CNS, the activation of IFN-I signaling in astrocytes suggests that IFN-I signaling might not be directly activated *via* HIV infection in microglia but through secondary bystander effect. Together, scRNA-seq analyses supported our proteomic data of persistent IFN-I signaling in the brain, which could occur in astrocytes during acute HIV infection and then in both astrocytes and MG during chronic infection of the brain on ART.

### IFN-I activation and HIV persistence in the brains of PWH on ART

**Fig 8:**
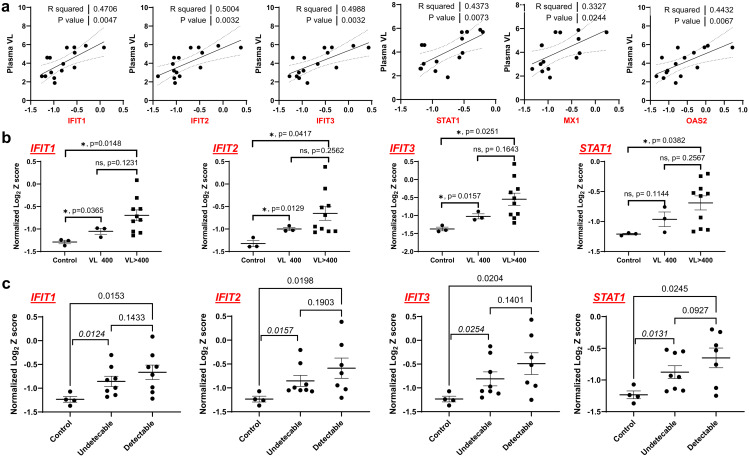
ISG induction is associated with HIV viral loads in HAND brains. A The association of ISG expression with plasma viral loads in PWH on ART. B The expression of ISGs in HAND brains from PWH with VL > 400 copies/mL or VL < 400 copies/mL. Statistics were analyzed by the Two-way ANOVA. C The expression of ISGs in HAND brains from PWH with detectable or undetectable HIV RNA. Statistics were analyzed by the Two-way ANOVA, in which the difference between control brains and brains with undetectable HIV RNA was determined using the two-tailed Welch’s T-test.

As the first line of antiviral defense, IFN-I signaling plays a central role in driving anti-viral response by modulating ISG expression in immune and non-immune cells [34]. HIV and SIV infection can trigger IFN-I signaling upregulation during both acute and chronic phases of infection [34–37]. ISG expression remained higher in HAND brains, which may reflect the host’s efforts to control HIV replication within CNS cells. Nevertheless, the persistent elaboration of IFN-I signaling during chronic HIV infection may contribute to immune activation, the interruption of the unique immune homeostasis environment within the brain, and the prediction of HAND progression. To this end, we decided to further analyze the interplay between HIV persistence and sustained IFN-I activation in the brains with suppressive ART. Virologic parameters and their relationship with the IFN-I pathway were studied in the frozen brain tissues of the donors from NNTC. Among the 27 cases of PWH on ART, 20 had VL > 400 near the time of death (74%), 5 had VL ≤ 400 copies/ml (19%), and 2 cases did not have available VL data (7%). We observed that ISG levels were increased in brain tissues, which were weakly associated with plasma HIV RNA levels (R-squared = 0.33-0.5) (Fig 8A). We next compared ISG levels between the HAND groups with VL > 400 (n = 9) and VL ≤ 400 copies/ml (n = 3) as well as with the HIVneg control group (n = 3). Surprisingly, ISG upregulation was detected in both the VL^high^ group and the VL^low^ group, compared with HIVneg controls. Of note, there was no significant difference in IFN-I activation between the VL^high^ and VL^low^ groups (Fig 8B).

Considering that the peripheral plasma VL may not accurately reflect the status of viral persistence within the CNS at the time of death and that many CSF viral loads were not available (S2, S3 Tables), we decided to directly measure the HIV RNA expression in brain tissues as a proxy of residual CNS viral replication. Among them, HIV RNA remained undetectable in 8 out of 16 available HAND brain samples determined by RT-qPCR. The lack of HIV transcripts in the brains of these 8 undetectable donors was further confirmed with a highly sensitive and validated HIV-specific digital droplet (dd) PCR [6], which showed that HIV levels were under the detection limitation (<50 copies/gram tissues). With these tissue viral load data, we found that ISGs remained significantly upregulated in the HIV-detectable group, compared with the HIVneg control group. No significant difference in ISG expression was observed between the HAND brains with and without detectable HIV RNA (Fig 8C). Of note, there was a significant difference in ISG expression between the HIVneg controls and samples with undetectable VL (Fig 8C, with Welch’s 2-tailed T-test). Together, these findings indicate that limiting local HIV replication by effective ART is not sufficient to eliminate persistent IFN-I activation in the brain. This is consistent with previous observations of persistent macrophage/microglia activation even under long-term viral suppression with ART [3–5,29,38,39]. While we cannot exclude the possibility that residual HIV expression undetectable by ddPCR contributes to the low levels of IFN-I activity during ART, or that limitations in anatomic sampling may miss regions that disproportionately contribute to the total CNS reservoir and drive a broader IFN signature, our data suggest that IFN-I signaling activation may not be solely due to persistent HIV infection. Some of the other mechanisms must play an essential role in persistent neuroinflammation.

### HERV-W1 Env induction may serve as a sustained stimulus to trigger the persistent IFN-I activation in HAND brains

Long-term effective ART neither eradicates immune activation nor reduces the prevalence of subtle HAND [3,4,38,40]. Therefore, we hypothesize that some non-HIV viral factors must exist to stimulate and maintain immune activation in the brains of PWH with HAND. HIV co-infections may contribute to immune activation [41]. Co-infection with the hepatitis C virus (HCV) or cytomegalovirus (CMV) could be an important risk factor for cognitive impairment [42,43]. It may induce inflammatory responses within the CNS, causing neurological injury^40^. In this study, 5 HAD donors and 2 MND donors had an HCV or CMV co-infection (S2, S3 Tables). However, we did not find any difference in ISG expression between the HAND brains with and without co-infection (S9 Fig). These data suggest that HIV co-infection with other exogenous viruses is not a major driver in the persistent IFN activation in the HAND brains. Some endogenous viral element induction may be involved.

**Fig 9:**
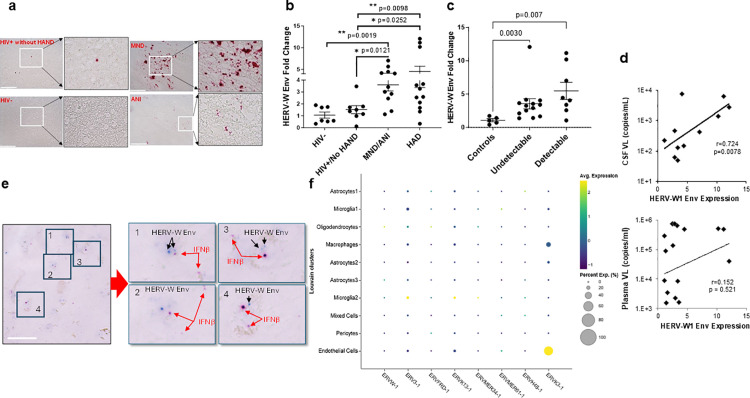
HERV-W1 Env pathogenic expression within the CNS during HAND. A HERV-W1 Env was detectable in HAND brains but not HIV+HAND- or HIVneg brains. scale bar, 100 µm. B HERV-W1 Env expression was associated with HAND and the HAND disease progression. HERV-W1 Env in the brain (deep WM) of indicated groups of PWH was assessed by RT-qPCR. The fold change of HERV-W1 Env was calculated after it was normalized to GAPDH and HIV-negative controls. *p < 0.05; **p < 0.001, compared with HIV- group; #, *p* < 0.05, compared with HIV+ group no HAND. C HERV-W1 Env was induced in HAND brains from PWH with either detectable or undetectable viral loads. D HERV-W1 Env expression was positively correlated with CSF VL but not plasma VL. also detectable in HAND brains with undetectable tissue HIV RNA. E Dual RNAscope analysis showed HERV-W1 Env (blue) and IFNβ (Red) were closely localized with each other. scale bar, 50 µm. F HERV-W1 Env expression in CNS cells in the brain of PWH on suppressed ART. The size of each circle represents the percentage of cells in the cluster where the gene is detected.

The pathogenic activation of HERVs was reported to be able to trigger immune activation in the brain [44–48]. To this end, we analyzed HERV-W1 Env transcripts in the brain tissues from PWH with HAND on ART in the same snap-frozen brain tissues from NNTC. HERV-W1 Env transcripts were detectable in all these tissues where MND/ANI had more HERV W1 Env than HAND neg or HIVneg controls (Fig 9A). HERV W1 Env expression was further quantitated by RT-qPCR. We found that the upregulation of HERV-W1 Env in the brain tissues was significantly associated with the presence of HAND (both HAD and MND) (Fig 9B). The HERV-W1 Env expression remained high in brains from PWH with HAND even when plasma VL was low (<400 copies/ml) (Fig 9C). Importantly, while there was no difference in HERV-W1 Env expression between the brain tissues with or without the detection of HIV RNA, HERV-W1 was significantly higher in HAND brain tissues with undetectable brain VL, compared with control brains without HAND (Fig 9C). Of note, HERV-W1 Env expression was positively correlated with HIV in the CSF viral loads (VL) but not the plasma VL (Fig 9D). In the brain tissues of PWH with HAND, HERV-W1 Env and IFNβ were closely localized with each other (Fig 9E) while IFNα was undetectable, consistent with the observations that HERV-W1 Env activation induces IFN-I activation in the CNS^28,^ [44]. To investigate which cells harbor HERV-W1, scRNA-seq was leveraged to analyze HERV expression. Up to 8 members of HERV were able to be detected in the brain on ART. While the expression of all these HERVs was relatively low, HERV-W1 (ERVW-1) was detectable in oligodendrocytes and astrocyte subset 3, but less in MG. Interestingly, MG2 expressed ERVMER34–1 and ERVH48–1, and MG1 had a reduced expression of ERVMER34–1 and ERVH48–1, both of which were known to directly inhibit HERV-M1 Env signaling [49]. Thus, the inhibition of ERVMER34–1 and ERVH48–1 may promote HERV-W1/syncytin-1 function in MG1. These observations support the previous studies, which reported that HERV-W1 Env expression in the glial cells activates the innate immune response to induce IFNβ and other inflammatory genes like TNFα [50,51].

**Fig 10:**
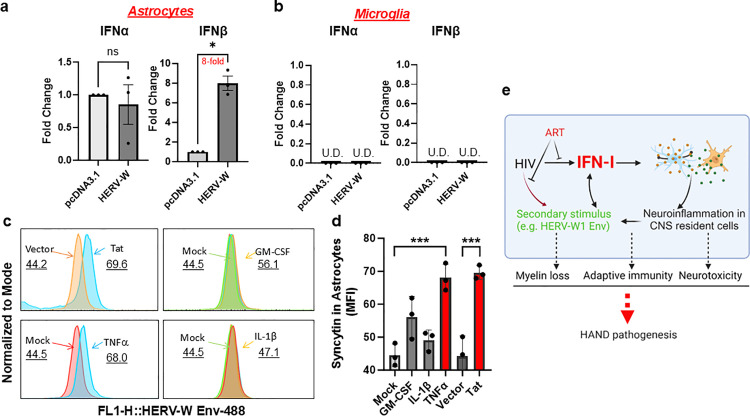
HERV-W1 Env induces IFN-I production in astrocytes. One microgram of empty vectors or HERV-W1 Env plasmids was transfected into human primary astrocytes (A) or microglial cells (B) in 6-well plates. Forty-eight hours post-transfection, cells were collected for the extraction of total RNA, which was subjected to the RT-qPCR analysis of the IFNα or IFNβ expression. The fold change of IFN-I signaling genes was calculated after they were normalized to the internal controls and HIVneg controls (n=3). *, *p*<0.05; U.D., undetectable. C-D HIV Tat expression or inflammatory cytokine TNFα stimulation induced HERV-W1 Env expression in the primary human astrocytes, but not in the cells that were treated with GM-CSF or IL-1β. HERV-W1 Env expression was measured using flow cytometry. ***, *p*<0.001, One-way ANOVA. E A self-fueling IFN-I activation induces persistent neuroinflammation in HAND despite ART. This is sustained by the residual HIV infection and a secondary stimulus, such as HERV-W1 Env or pro-inflammatory cytokines. The chronic activation of the IFN-I pathway promotes and maintains neuroinflammation, which produces neurotoxins, including ISGs. The abnormal expression of HERV-W1 Env also contributes to HAND pathogenesis, independent of ART. The figure was created using BioRender.

To provide further evidence for HERV-W1 Env’s role in directly activating IFN-I in CNS cells without HIV infection, we transiently transfected the pcDNA3.1 control or pcDNA3.1-HERV-W1 Env plasmids into the primary human astrocytes or microglia. We found that IFN-I cytokine IFNβ, but not IFNα, was induced in the primary astrocytes, which was upregulated 8-fold, compared with the empty vector control (Fig 10A). This is consistent with the previous report^47^. Surprisingly, neither IFNs was induced in the human primary MG (Fig 10B). This is in line with our scRNA-seq analysis in astrocytes in our MCO model, in which IFN-I activation largely occurred in astrocytes but not in MG (S9F Fig). Lastly, either HIV Tat expression or inflammatory TNFα stimulation significantly induced the expression of HERV-W1 Env in astrocytes but not in wells that were treated with GM-CSF or IL-1β (Fig 10C, 10D), consistent with previous reports [37,45,47,52].

Together, these observations support that HERV-W1 reactivation by HIV Tat contributes to IFN-I activation in the brain astrocytes. Since ART is designed for HIV replication but not HERV-W1 expression, unresolved HERV-W1/IFN-I activation loop may persist in neuroinflammation as an additional stimulus for HAND pathogenesis in PWH on ART.

## Discussion

In this study, we discovered a persistent immune activation pathway in HAND brains despite ART. Among the 21 upregulated immune activation pathways, the IFN-I pathway was the most dominant upregulated signaling in HAND brains, even in those with an effective ART. This indicates that ART alone failed to resolve the persistent IFN-I activation in the brain, possibly in some of the brains we have tested. Consistent with our proteomic analyses of upregulated ISG expression, we detected elevated levels of IFNβ transcripts, to a lesser extent IFNα, in HAND brains. Our scRNA-seq transcriptome data further validated such a signature of IFN-I activation in the brain MG from ART-suppressed PWH.

Unlike the well-documented IFN-I activation in ART-free HAND brains [35,53,54], IFN-I activation was less characterized during chronic viral infection under ART. Followed by the successful viral control of effective ART, HIV-triggered IFN-I activities should have been diminished. However, we and others have shown that chronic IFN-I activation and glial activation persist in the brains of PWH [3–5,40,55], even in HAND brains when HIV expression was suppressed by ART to undetectable levels. The residual HIV expression may be under the detection limitation of qRT-PCR. However, HIV transcripts were even undetectable by the extremely sensitive ddPCR assay. This may argue for the existence of viral transcripts in 8 of those brains from PWH, supporting the existence of true undetectable HIV RNA in ART-suppressed brains, where IFN-I remained persistent.

IFN-I was activated in the astrocytes of brain organoids after acute HIV infection, but it persists in MG isolated from PWH receiving suppressive ART. Notably, acute HERV-W1 expression directly induced IFNβ expression in astrocytes. Therefore, the pathogenic HERV-W1 Env expression in the HAND brain may serve as a secondary but HIV-independent stimulus for the persistent IFN-I activation in HAND brains. Thus, limiting HIV infection by ART alone won’t be sufficient to prevent immune activation, IFN-I signaling persistence, or HERV-W1-associated HAND pathogenesis in the brain^44^. These observations link pathogenic positive feedback signaling to the sustained persistent IFN-I immune activation in the human brain (Fig 10E). Nevertheless, there is a limitation in this study due to different models being used, such as tissues from NNTC, the Last Gift cohort, and HIV-infected brain organoids. Future study is warranted to further validate these important findings.

The activation of IFN-I was originally thought of as an anti-viral innate immunity during acute viral infection. However, *in vivo* studies have shown that IFN-I’s innate immunity is usually ineffective in suppressing HIV infection under chronic viral infection. This is due to the viral factors that counter the anti-viral activities of ISGs and immune suppression [56]. In this study, we discovered a strong association between the abundance of ISGs and their related adaptive immune proteins during chronic viral infection receiving suppressive ART, pointing to an important role of IFN-I signaling in coordinating both innate and adaptive immune responses to damage the CNS. This is similar to the role of IFN-I in the periphery [57]. During chronic infection, IFN-I signaling drives exhaustive activation and promotes viral persistence [56]. As evidenced in Sooty mangabeys and African green monkeys [58], anti-viral IFN-I responses are mounted during acute viral infection; however not sustained but resolved in the chronic phase, contributing to their nonpathogenic outcomes. In contrast, pathogenic SIVmac infection in rhesus macaques induced hyperactive IFN-I signaling [59]. Similarly, within the CNS, the elevated IFN-I signaling may eventually suppress the immunity in the CNS, promote infection in the brain, and induce neuroinflammation and cognitive impairment [25]. This is because the persistent IFN-I activation in the brain can be highly neurotoxic and has been associated with a wide range of neurological diseases, which have symptoms, including cognitive and motor dysfunction, found in HAND. This has also been proven in neurological diseases. In contrast, IFNAR1 deletion or anti-IFNAR1 neutralization resulted in the downregulation of proinflammatory cytokines and improved symptoms [60]. The adverse effects from IFN therapy also reveal a causal contribution of increased IFN-I signaling to neuropathogenesis [60]. For instance, a long-term or high dosage of IFNα therapy resulted in cognitive dysfunction, including cognitive slowing, disorientation, memory deficits, and impaired motor and executive function [61].

Interestingly, unlike IFNα, several studies showed that IFNβ appears to temporarily protect the brain, exerts anti-inflammatory effects, and has fewer adverse effects than IFNα(25, 53). IFNβ has been used for the treatment of MS [62]. During the acute SIV infection, IFNβ was protective in viral infection [63]. IFNβ was also upregulated in HIVgp120 transgenic mice [64]. It may not be surprising that IFNβ was upregulated and protective to combat HIV infection since, in both HIV infection-related models, the infection or the mimics of the infection was in the acute, but not chronic, phase of infection. Our current studies focused on HIV chronic infection in PWH on ART and demonstrated an upregulation of both IFNα and IFNβ in HAND brains, where IFNβ was about 2-fold higher than IFNα. Together, these data show that IFN-I signaling plays a unique role in chronic HIV infection, which could be detrimental to the CNS if IFN-I persists, and/or if it is not properly resolved. In line with this, the blockade of IFNβ, but not IFNα, controls persistent viral infection to accelerate virus clearance [65].

IFNβ transcripts, and to a lesser extent IFNα, were much higher in HAND brains than the HAND negative brains. Since neurons are not permissive to HIV infection, the neuronal damage detected in the HAND brain must be indirectly induced by the effects of neurotoxins produced in the CNS cells following IFN-I activation [60]. While most ISGs are essential in restricting acute viral infection, persistent ISG expression could be neurotoxic during chronic viral. For example, ISG15 upregulation leads to cellular necrosis and the interruption of the blood-brain barrier, leading to neuronal damage and neurologic disorders [66]. The level of ISG15 is significantly higher in the gray matter of both HAD and MND/ANI brains, potentially associated with brain damage. ISG15 was enriched in neurons and OPCs. The latter are known glia subset cells that are essential in oligodendrocyte development. OPC inflammation will cause a loss of myelination and the subsequent impairment of neurological functions [67]. Emerging data also support the role of ISG15 in blocking HIV release [68] and viral latency [69,70]. Therefore, ISG15 may serve as a novel biomarker and therapeutic target for HAND, which is worth further investigation.

Several studies have reported that in the periphery, anti-viral IFN-I signaling plays a unique role in maintaining viral latency in HIV-infected humanized mice and SIV-infected NHPs on suppressive ART [56,71,72]. IFN-I activation suppresses viral replication while inhibiting antiviral IFNβ enhances HIV replication in myeloid cells [73], Of note, it is IFNβ, but not IFNα [74], that is critical for HIV latency *in vivo*, suggesting that IFNβ plays a unique role in maintaining HIV latency. *In vivo*, the IFN-I cytokine IFNβ restricts SIV infection and inflammation in the brain during acute SIV infection in NHPs [75]. Conversely, knocking out the IFN-I receptor increases HIV expression and induces MG activation, leading to neuropathogenesis in EcoHIV-infected mice [76].

Except for HIV infection, two other non-HIV viral factors participate in the induction of IFN-I in the HAND brains: 1) co-infection with other exogenous viruses; and 2) the reactivation of endogenous retroviruses and/or their viral products following the initial HIV infection. However, co-infection of HIV with exogenous viruses had a minimal impact on IFN-I activation in the HAND brains. Instead, endogenous HERV-W1 Env was highly induced and associated with IFN-I signaling in the HAND brains. HERV-W1 activation has been documented in many neuroinflammatory diseases, including MS [47,48,51]. The latter shares the common feature of persistent immune activation in HAND with the release of proinflammatory cytokines, chemokines, and neurotoxicity in the brain [77]. The expression of HERVs is quiescent or tightly controlled in the quiescent state unless reactivated by the infection of exogenous viruses, including HIV [36,37] or indirect induction following neuroinflammation [45,47,52,77]. Previous studies reported that by directly activating the HERV promoter [52], TNFα and IFN-I can activate HERV-W1 expression. In turn, HERV-W1 Env can activate IFN-I through the linc0193/cGAS axis [50], thereby creating a positive feedback loop of IFN-I activation. We found that HIV Tat or inflammatory cytokine can directly activate HERV-W1 Env. Of note, other family members of HERV also expressed in MG, indicating their possible roles in neuroinflammation.

It has been reported that HERV-W1 Env (i.e., syncytin-1) was elevated in glial cells in patients with acute MS, which exhibited cytotoxicity to oligodendrocytes and caused cell death and demyelination [78]. Interestingly, besides HERV-W1, many other HERV family members were induced in the brains of PWH on ART, including HERV-W1. Unfortunately, except for HERV-W1, the study of other HERV family members in HIV infection is limited due to their similar genome sequences. HERV-W1 expression also induced IFNβ, increased neuronal apoptosis, and played a role in Schizophrenia pathogenesis [50]. *In vitro*, HERV-W1 Env expression in astrocytes induced the release of redox reactants, which were cytotoxic to oligodendrocytes [78]. As aforementioned, oligodendrocyte damage causes axonal demyelination and neuronal injury, leading to neurological disorders. MS and HAND have common functional deficits upon the loss of oligodendrocytes and myelin sheath. Conversely, the treatment with antibodies to HERV-W1 Env rescued myelin expression [79]. We found that the pathogenic activation of HERV-W1 Env was linked to HAND even in the brains of PWH on suppressive ART. Thus, HERV-W1 Env activation may play a role in HAND pathogenesis. Of note, many other HERVs are also expressed in CNS cells on ART, indicating that some other HERVs could be involved in HAND pathogenesis. Unfortunately, approaches to study these HERVs are largely unavailable due to their genome similarity to host sequences and their highly variable nature [80], which is worthy of further investigation in the future when appropriate biochemistry and molecular biology tools exist in the future.

Together, our study discovers pathogenic positive feedback signaling that is linked to the sustained persistent IFN-I immune activation in the human brain on ART. Since it can be HIV-independent and ART-insensitive, residual but persistent IFN-I activation may self-fuel neuroinflammation in the brain, thereby playing a pivotal role in mild and subtle cognitive alterations in HAND on ART (Fig 10E).

## Materials and methods

### Ethics statement

Participants with HIV on ART were from the Last Gift cohort and the National Disease Research Interchange (NDRI). The Last Gift program enrolled altruistic, terminally ill PWH who had a diagnosis of HIV with < 6 months to live without CNS malignancy or immune checkpoint therapy. Formal consent was obtained in writing during autopsies. The IRBs of the University of California, San Diego (IRB no. 160563) and the National Disease Research Interchange approved the studies.

**Brain tissues** Frozen brain tissue samples (WM from deep WM of the frontal lobe and/or frontal cortex) were collected from 27 HIV+ Individuals and 9 HIV- individuals between 1999 and 2009 from the National NeuroAIDS Tissue Consortium (NNTC)(Manhattan HIV Brain Bank, National Neurological AIDS Bank, California NeuroAIDS Tissue Network, and Texas NeuroAIDS Research Center). They were stored at − 80 °C. HAND clinical diagnoses and clinical data were also collected through NNTC (S1 Table).

### Tandem mass tag quantitative proteomics

*Protein extraction and digestion* Tissue pieces were lysed in a lysis buffer (8 M urea, 50 mM tris-HCl, pH 8.0) with sonication (1s on 1s off, 20 s, twice). Centrifugation was performed for 20 min at 16,000 g at 4oC and the supernatant was collected. Proteins were reduced by 5 mM DTT (dithiothreitol) for 30 min, alkylated with 15 mM IAA (iodoacetamide) for 30 min in the dark. The solution was diluted with buffer 25 mM Tris (pH 8.0), and 1mM CaCl2 to make the final urea concentration 2 M. Tryptic digestion was performed with a ratio of 1:100 (trypsin: protein) for 12–16 hours at RT.

*Peptide labeling and mass spectrometry analysis* Peptides were cleaned with homemade C18 stage tips and the concentration was determined (Peptide assay, Thermo 23275). 50 µg each was used to label with isobaric stable TMT following manufacturer instructions. The mixture of labeled peptides was fractionated into 10 fractions on the C18 stage tip with buffer 10 mM Trimethylammonium bicarbonate (TMAB), pH 8.5 containing 5–50% acetonitrile. The peptides were dried and dissolved in 0.1% formic acid. 0.5 µg of each fraction was analyzed on a Q-Exactive HF-X coupled with an Easy nanoLC 1200 (Thermo Fisher Scientific, San Jose, CA). The peptides were loaded onto an Acclain PepMap RSLC C18 Column (150 mm × 75 μm ID, C18, 2 μm, Thermo-Fisher). Analytical separation of all peptides was achieved with a 130 min gradient. Multiple linear gradients of 5–10% buffer B over 5 min, 10–31% buffer B in 100 min and 31–75% in 15 min were executed at a 300 nl/min flow rate. This was followed by a ramp to 100%B in 1 min and a 9-min wash with 100%B. Buffer A was aqueous with 0.1% formic acid and buffer B was 80% acetonitrile and 0.1% formic acid. LC-MS experiments were also carried out in a data-dependent mode using full MS with a resolution of 120,000. This was followed by a high energy collision-activated dissociation-MS/MS of the top 20 most intense ions with a resolution of 45,000. High energy collision-activated dissociation-MS/MS was used to dissociate peptides at a normalized collision energy of 32 eV in the presence of nitrogen bath gas atoms. The dynamic exclusion was 45 seconds.

*Raw proteomics data processing and analysis* Peptide identification and quantification with TMT reporter ions were performed using the MaxQuant software version 1.6.10.43’s (Max Planck Institute, Germany) built-in search engine, Andromeda. Protein database searches were performed against the UniProt human protein sequence database (UP000005640). A false discovery rate (FDR) for both the peptide-spectrum match (PSM) and protein assignment was set at 1%. Search parameters included up to two missed cleavages at Lys/Arg on the sequence, oxidation of methionine, and protein N-terminal acetylation as a dynamic modification. Carbamidomethylation of cysteine residues was considered a static modification. Peptide identifications are reported by filtering reverse and contaminant entries and assigning them to their leading razor protein. Data processing and statistical analysis were performed on Perseus (Version 1.6.0.7). Protein quantitation was performed on biological replicates and two-sample t-test statistics was used with a p-value of 5% to report statistically significant protein abundance fold-changes. Tissue gene analysis was performed by ARCHS4 in Enrichr. The analysis of canonical pathways and functions was performed with the QIAGEN Ingenuity Pathway Analysis (IPA) and the Elsevier Pathway Collection in Enrichr. Synapse structure and function were analyzed by SynGo Gene Ontologies [14]. The mass spectrometry proteomics data have been deposited to the ProteomeXchange Consortium via the PRIDE partner repository with the dataset identifier PXD042550.

#### MAP2 immunochemistry.

Brain tissues were fixed in 10% formaldehyde, embedded in paraffin, and then sectioned into 6 μm-thick slices. Histological sections underwent deparaffinization using xylene baths, followed by rehydration through ethanol. Subsequently, the slides were exposed to hydrogen peroxide at room temperature for 10 minutes, followed by boiling (100°C) in 1X RNAscope target retrieval buffer for 30 minutes. Then, the slides were treated with 100% ethanol, followed by protease plus at 40°C for an additional 30 minutes. A hydrophobic barrier was created using a hydrophobic barrier pen to facilitate the efficient application of reagents. The slides were blocked with a mixture of 15% goat serum and 1% FcR human block in TBST for 45 minutes. The primary MAP2 antibody (diluted 1:100 in PBST) was incubated overnight at 4°C. After three washes with 1XTBST, a biotinylated secondary antibody diluted at 1:800 was incubated for 30 minutes. Following a single 5-minute wash, slides were incubated with peroxidase streptavidin at a 1:500 dilution in TBST. After a final 5-minute wash, slides were exposed to ImmPACT DAB Peroxidase substrate (Cat. No SK-4105) at room temperature for 2 minutes to develop the staining. The reaction was stopped by rinsing with distilled water. The image was further analyzed by ImageJ.

**Transfection of HERV-W1 Env into CNS cells** Human primary microglia or astrocytes were transfected with 1 μg pcDNA3.1 or pcDNA3.1-HERV-W1 Env plasmids. Forty-eight hours after transfection, cells were collected for the extraction of total RNA. Then, the RNA was pretreated with DNase. Then, cDNA was generated using SuperScript IV Reverse Transcriptase (Invitrogen). IFN-I signaling gene transcription was determined by qPCR, where TBP served as an internal control.

**RT-qPCR to quantify HIV and IFN-I signaling transcripts** Tissue RNA was isolated from myeloid cells or 100 mg of tissues using a RNeasy kit (Qiagen) and treated with 1 unit of DNase (Life Technologies) for 30 min at 37°C before reverse transcription into cDNA. Triplicate PCR reactions were performed for each sample. The HIV tissue residual RNA was amplified with gag-PCR [81]. HIV transcripts in the tissues were also quantified by Digital droplet PCR (ddPCR) and performed in a Bio-Rad QX-100 system as described previously [6]. Genes from the IFN-I signal pathway, other immune responses, and non-immune related pathways were amplified using the commercially available primer-probe sets (ThermoFisher).

### Microglia-containing cerebral organoid model of HIV infection and single-cell RNA-seq analysis of gene transcriptome in derived CNS cells

MCOs induced from iPSC cells of healthy donors were cultured according to our established protocol 33. At 7 days postinfection with JR-CSF-GFP virus, MCOs were washed with 1x DPBS and dissociated in 2 mL of Accutase (Sigma) containing 0.4 U/µL Dnase I (QIAGEN) at 37°C for 30 min, followed by two rounds of filtration with 40 μm strainer and rinsing with 3 ml MCO culture media. Dissociated cells were harvested by centrifugation at 300 g for 5 min at 4°C and resuspended in 1 mL of chilled 1x DPBS. Cell viability was assessed by Trypan blue staining and counted using an automatic cell counter (Countess, ThermoFisher, Inc.). Freshly prepared cells were sent for sequencing via a 10x genomics system (SingulOmics, NY). Raw data were analyzed by the Cellranger pipeline using human GRCh38 and JR-CSF-GFP as references. The featured barcode matrix was analyzed using the Seurat package [82] (see below) with a clustering resolution at 0.1 for 6 cell types identified by known specific markers. Genes related to IFN-I signaling were used to identify cellular distribution. The dataset will be provided upon request.

#### Single-cell RNA-seq analysis of gene transcriptome in CNS cells isolated from PWH on ART.

a. *Construction of gene expression library and sequencing.* Fresh brain tissue pieces were obtained during rapid autopsies through the “Last Gift” cohort [7]. CNS cell isolation was performed with a recently validated protocol [6]. In brief, the brain tissue pieces were dissociated by performing both mechanical and enzymatic dissociation steps. A personal gradient step was used to remove the myelin debris, and the single CNS cell suspension was generated. Brain myeloid cells were further purified via CD11b selection as described before [6]. The isolated brain cells were then used to generate scRNA-seq libraries using the Chromium Next GEM Single Cell 3’ reagent dual index kit v3.1 following manufacturer’s protocol (CG000315 • Rev E). Briefly, diluted cell suspensions (>90% viability) with a targeted recovery of 10000 cells were barcoded using GEM (Gel Beads-in-emulsion) beads. A Chromium Single Cell Controller (10xGenomics, Pleasanton, CA) was used to generate single-cell GEM suspensions (GEMs). Reverse transcription of the GEMs produces 10x Barcoded, full-length cDNA from poly-adenylated mRNA. Barcoded cDNA was pre-amplified to facilitate a gene expression (GEX) library construction. cDNA amplification, enzymatic fragmentation followed by end Repair, A-tailing, adaptor Ligation, and PCR were performed to incorporate P5, P7, i7, and i5 sample indices, and TruSeq Read 2 (read 2 primer sequence) for gene expression libraries. Silane magnetic beads and SPRIselect (Beckman Coulter) were used for the purification and size selection of the barcoded products from the post-GEM-RT reaction mixture and gene expression libraries, respectively. The size selected libraries were quantified using an Agilent Tapestation 4200 and the Qubit dsDNA High Sensitivity Assay Kit (Invitrogen, #Q33230). Pooled samples of RNA libraries were sequenced using paired-end, dual-index sequencing on a NextSeq 2000 instrument (Illumina). The read format for scRNAseq was: Read 1–28 cycles; Read 2–90 cycles; i7 - 10 cycles; i5 – 10 cycles. Obtained paired-end reads of pooled libraries were demultiplexed before read mapping.
b. *scRNA-Seq read mapping and counting.* Cellranger v4.0, developed by 10X Genomics, Inc. (Pleasanton, CA, USA) was used for read mapping and counting of fastq raw data. The Genome Reference Consortium Human Reference 38 combined with the HIV genome reference was used for mapping with default parameters for CellRanger that account for MAPQ adjustment, examination of compatibility with the transcriptome, unique molecular identifiers (UMI) counting, and cell-calling. The output filtered_feature_bc_matrix folder, which contains the barcodes after cell-calling filtration, was used for downstream analyses.
c. *Identification of major cell clusters.* Parse Biosciences Trailmaker was used for scRNA-seq data processing. Before clustering, we set a series of criteria; the classifier filter was set at false discovery rate (FDR) at ≤ 0.01, cell distribution to filter out low-quality cells. Cells with transcript numbers lower than 200 genes were excluded. Next, cells expressing mitochondria genes more than 3% of the transcriptome were filtered out. The number of genes and transcript prediction interval was set at 0.99 and <0.0003. The doublet filter was set automatically to probability threshold 0.67. For data integration, Seurat was used with 2000 high variance genes for Principal Component Analysis (PCA) with the number of principal components set to default with variation explained. Furthermore, ribosomal, mitochondrial, and cell cycle genes were also excluded. Cells were embedded in UMAP minimum distance = 0.3, cosine metric, and projected onto two dimensions, and assigned clusters by Louvain clustering. Cell clusters were visualized in a two-dimensional UMAP space.
d. *Cell type identification.* The human brain is composed of a diverse array of cell types that vary in terms of their molecular and functional properties. Single-cell genomics such as single-cell RNA sequencing allows for a more comprehensive and detailed molecular characterization of these cell types by simultaneously measuring the expression of thousands of genes. By analyzing single-cell gene expression profiles, researchers can identify distinct cell populations and develop molecular signatures that define individual cell types. This has revolutionized our understanding of the diversity of cell types in the human brain and has helped to uncover new, previously unknown cell types. In this study, we used a human brain-specific scRNA-seq reference dataset from Darmanis et al [83] to identify human brain cells and representative markers. Using this reference, clusters were assigned to cell types using the MaqQuery wrapper function (Seurat R package 4.3.0 [82]).
e. *Enrichment Analysis.*

Ingenuity Pathways Analysis (IPA) was used to analyze the differentially expressed (DE) genes from the different clusters. DE genes were defined as log fold change -0.5 ≤ or ≥ 0.5, p < 0.05. The top enriched canonical pathways and diseases and functions were identified. For interferon signaling, pathways within the top 50 were considered significant.

f. Comparative gene expression. Finally, we compared the expression of the main genes of the inflammasome pathway (GO: 0061702) and cell death (GO: 0097194) in microglial and endothelial cells identified using the method described above.

The GEO accession number for the data deposit is GSE233717, or the dataset will be provided upon request.

**Statistical analysis** Changes in HAND groups compared with HAND negative controls were performed and analyzed using Prism GraphPad 9.1. The correlation was achieved through linear regression in Prism. Statistical significance was determined by ANOVA using multiple comparisons or by two-tailed student’s t-test between two groups. Data were presented as mean ± SEM.

## Supporting information

S1 Fig IFN-I signaling proteins are activated in the sub-regions of HAND brains. IFN-I signaling proteins IFIT1, IFIT3, MX1, ISG15, IFIT2, OAS2, and STAT1 were upregulated in the gray matter (GM) of HAND brains. The abundance of Z-scores of indicated proteins in HAND brains (n = 11) was compared with the scores in the control brains (HIV- control, n = 2; and HIV+HAND- controls, n = 2). The p-value was calculated by a two-tailed *t*-test in comparison with controls.
(PPTX)

S2 Fig Immune activation markers are upregulated in HAND brains. The normalized Z-score of the indicated immune activation marker proteins in the grey matter of HAND brains (n = 11) was compared to the Z-score from HAND control brains (n = 4, 2 HIV- and 2 HIV+HAND- brains). The *p*-value was calculated by a two-tailed t-test in comparison with controls.
(PPTX)

S3 Fig Association of IFN-I proteins with the adaptive immune response in HAND brains. ICAM protein expression was positively correlated with IFN-I signaling protein STAT1 (A) and IFIT3 (B), which was analyzed using linear regression. A similar analysis was performed between IFIT1 and a few down-regulated proteins essential for neuronal functions, including KLK6, CD9, and TSPAN8 (C).
(PPTX)

S4 Fig Expression of IFN-I genes in HAND brains. MX1, STAT1, and HLA-A mRNA expression was analyzed by RT-qPCR in the HAND brains (n = 14), compared with HIV-negative brains (n = 6) and HIV+ but HAND- brains (n = 7). The *p*-value was calculated using Welch’s ANOVA comparison with controls.
(PPTX)

S5 Fig The expression of ISGs in HAND brains with different peripheral viral loads. The expression of ISGs in HAND brains from PWH with VL > 400 copies/mL or VL < 400 copies/mL. Statistical differences were analyzed by Two-way ANOVA.
(PPTX)

S6 Fig Single-cell RNA-seq analysis to characterize CNS cells isolated from LG29 enrolled in the LAST GIFT program on ART. UMAP projection (A) and heatmap (B) showed the expression of the representative CNS cell biomarkers (for example, HEXB and TMEM119 for MG, GFAP for astrocytes, OLIG1 and OLIG2 for oligodendrocytes, CD68 and CD163 for macrophages, and ALCAM for pericytes).
(PPTX)

S7 Fig Single-cell RNA-seq analysis to characterize CNS cells isolated from N7, enrolled in NDRI, who, stopped  ART 7 days before the death. Cluster analysis (A) and heatmap (B) showed the expression of representative biomarkers (HEXB and TMEM119 for MG; CD163 and CD68 for macrophages) of CNS cells.
(PPTX)

S8 Fig Single-cell RNA-seq analysis of cell death/survival pathways in MG isolated from PLWH on ART. Analysis of disease and function pathways uncovered altered cell death, survival, and proliferation signaling in CNS cells in LG29 (A) and N7 (B) on ART. (C) A study of proliferation genes in the subsets of myeloid cells on ART discovered unique subsets of MG that may acquire proliferation advantages critical for them to serve as stable viral reservoirs on long-term ART.
(PPTX)

S9 Fig Single-cell RNA-seq analysis of IFN-I in our MCO model of mini-brains acutely infected by HIV. MCO was established and infected by HIV for 7 days. Then, MCO was collected, and the single-cell suspension was prepared for scRNA-seq analyses of CNS cells (A), IFN-I activation (B), and HIV transcripts (C).
(PPTX)

S10 Fig IFN-I signaling in the HAND brains from PWH with or without viral co-infection. The protein abundance Z-scores of IFN-I signaling genes in the WM of HAND brains were compared with the Z-scores of HAND brains co-infected with HCV or CMV, and a two-way ANOVA test was performed to calculate the *p*-value.
(PPTX)

S1 Table Characteristics of the study cohort. (PPTX)

S2 Table Characteristics of 19 PWH with HAND for the quantitative TMT proteomic analysis. (PPTX)

S3 Table Characteristics of 8 HIV+ but HAND negative PWH for proteomics and RT-qPCR analysis. (PPTX)

S4 Table Characteristics of 2 PWH on ART for scRNA-seq analysis. (PPTX)

S5 Table Characteristics of HIV-negative donors for proteomics and RT-qPCR analyses. (PPTX)

S6 Table HAND was associated with significant upregulation of 30 proteins. (PPTX)

S7 Table Biological pathways identified in HAND brains. (PPTX)

S8 Table Down regulation of 27 proteins in HAND brains. (PPTX)

S9 Table Down-regulated proteins are highly expressed in the CNS, based on ARCHS4 (All RNA-seq and ChIP-seq sequencing) tissue gene set analysis. (PPTX)

S10 Table Analysis of down-regulated proteins by SynGo. (PPTX)

S11 Table Down-regulated proteins are associated with neurological diseases. (PPTX)

S12 Table Non-immune related factors are regulated in the HAND brains. (PPTX)
