# Supplementary figures and images for: Persistent type I interferon signaling within the brain of people with HIV on ART with cognitive impairment
Source: PLoS Pathog. 2025 Aug 20;21(8):e1013411. doi: 10.1371/journal.ppat.1013411 (PMC12367146; doi:10.1371/journal.ppat.1013411)

## Slide 1
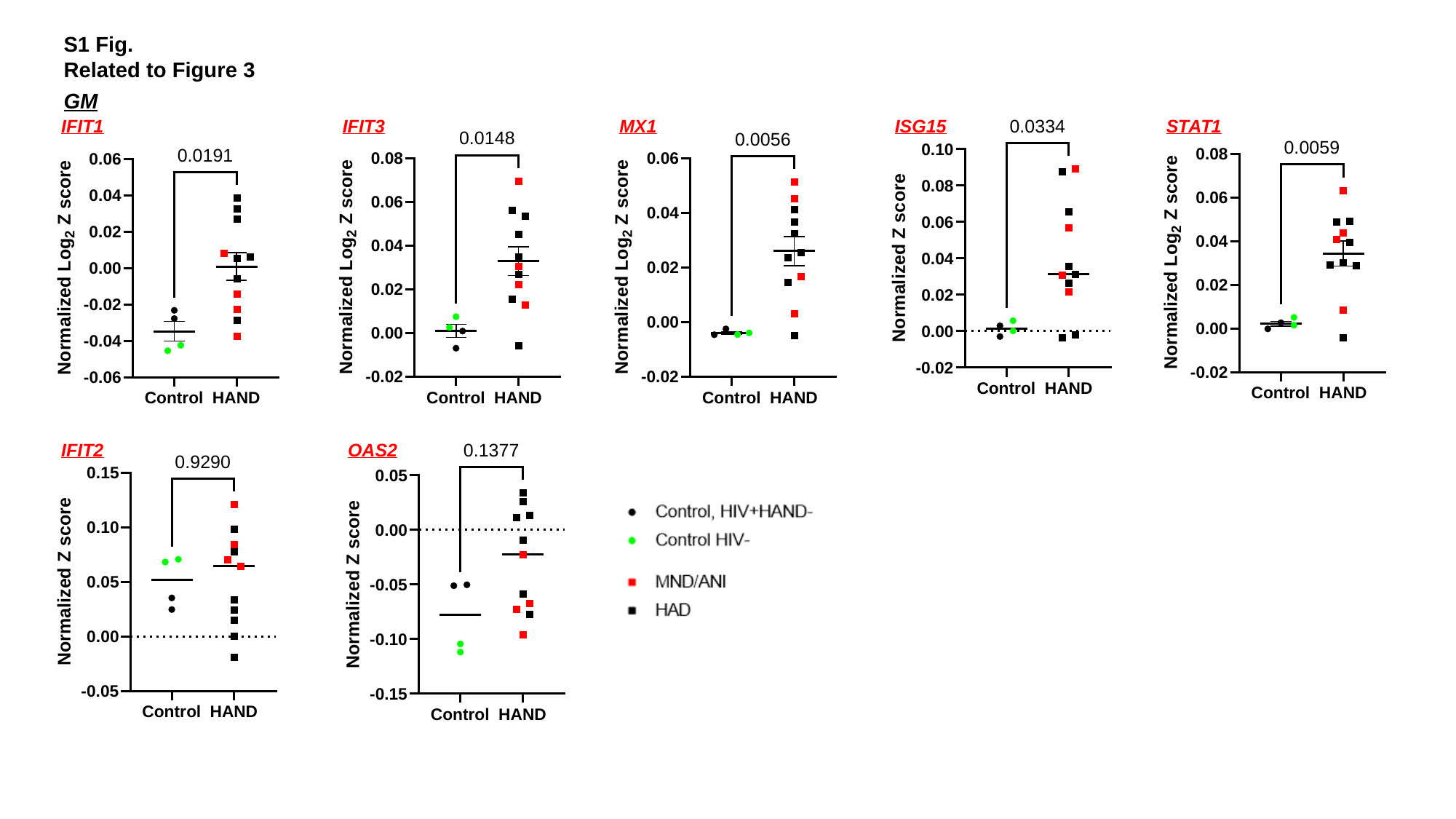

S1 Fig.
Related to Figure 3
GM

Supplement: S1 Fig — IFN-I signaling proteins IFIT1, IFIT3, MX1, ISG15, IFIT2, OAS2, and STAT1 were upregulated in the gray matter (GM) of HAND brains. The abundance of Z-scores of indicated proteins in HAND brains (n = 11) was compared with the scores in the control brains (HIV- control, n = 2; and HIV+HAND- controls, n = 2). The p-value was calculated by a two-tailed t-test in comparison with controls. (PPTX) [file ppat.1013411.s001.pptx]

## Slide 1
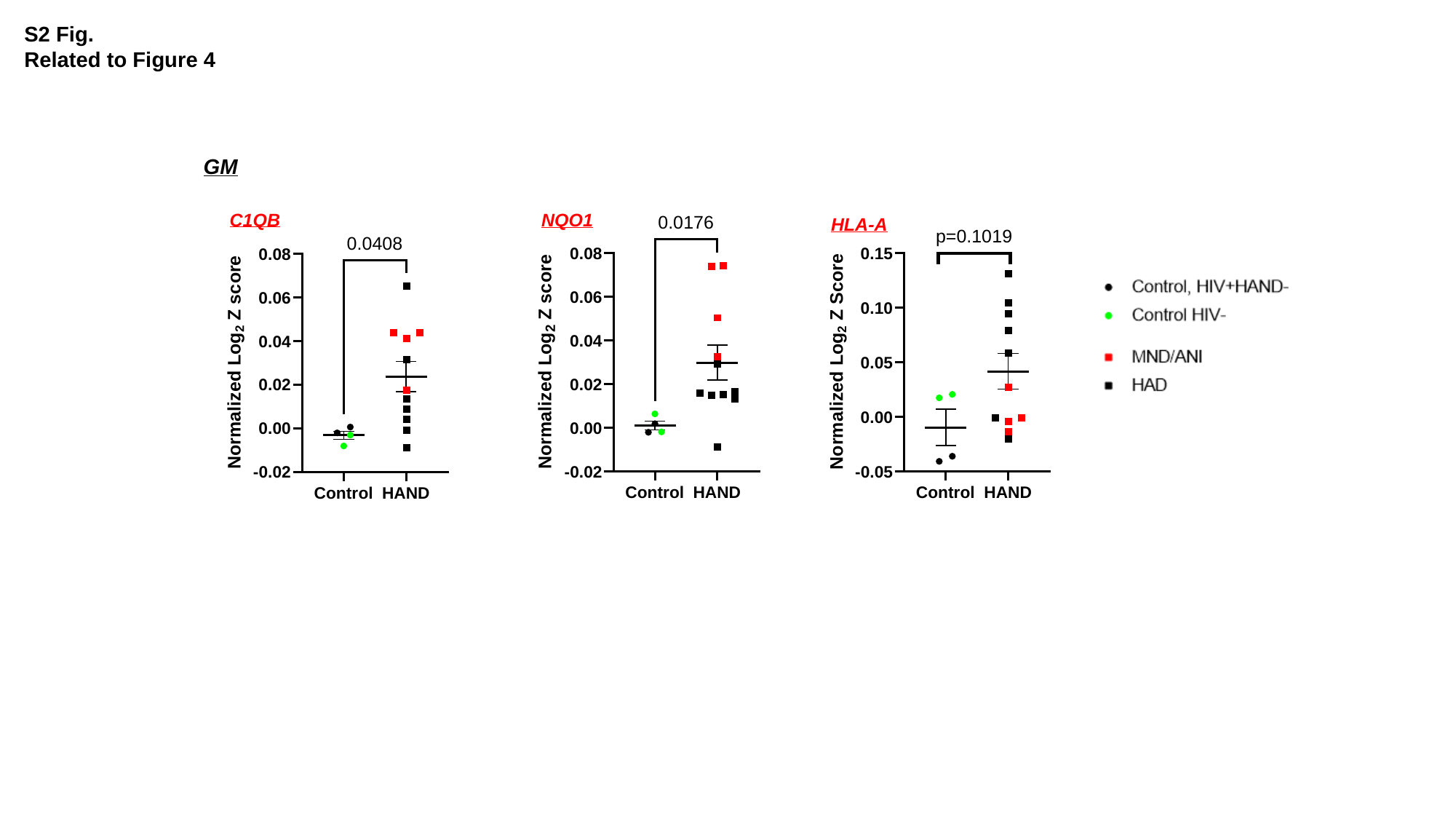

S2 Fig.
Related to Figure 4
GM

Supplement: S2 Fig — The normalized Z-score of the indicated immune activation marker proteins in the grey matter of HAND brains (n = 11) was compared to the Z-score from HAND control brains (n = 4, 2 HIV- and 2 HIV+HAND- brains). The p-value was calculated by a two-tailed t-test in comparison with controls. (PPTX) [file ppat.1013411.s002.pptx]

## Slide 1
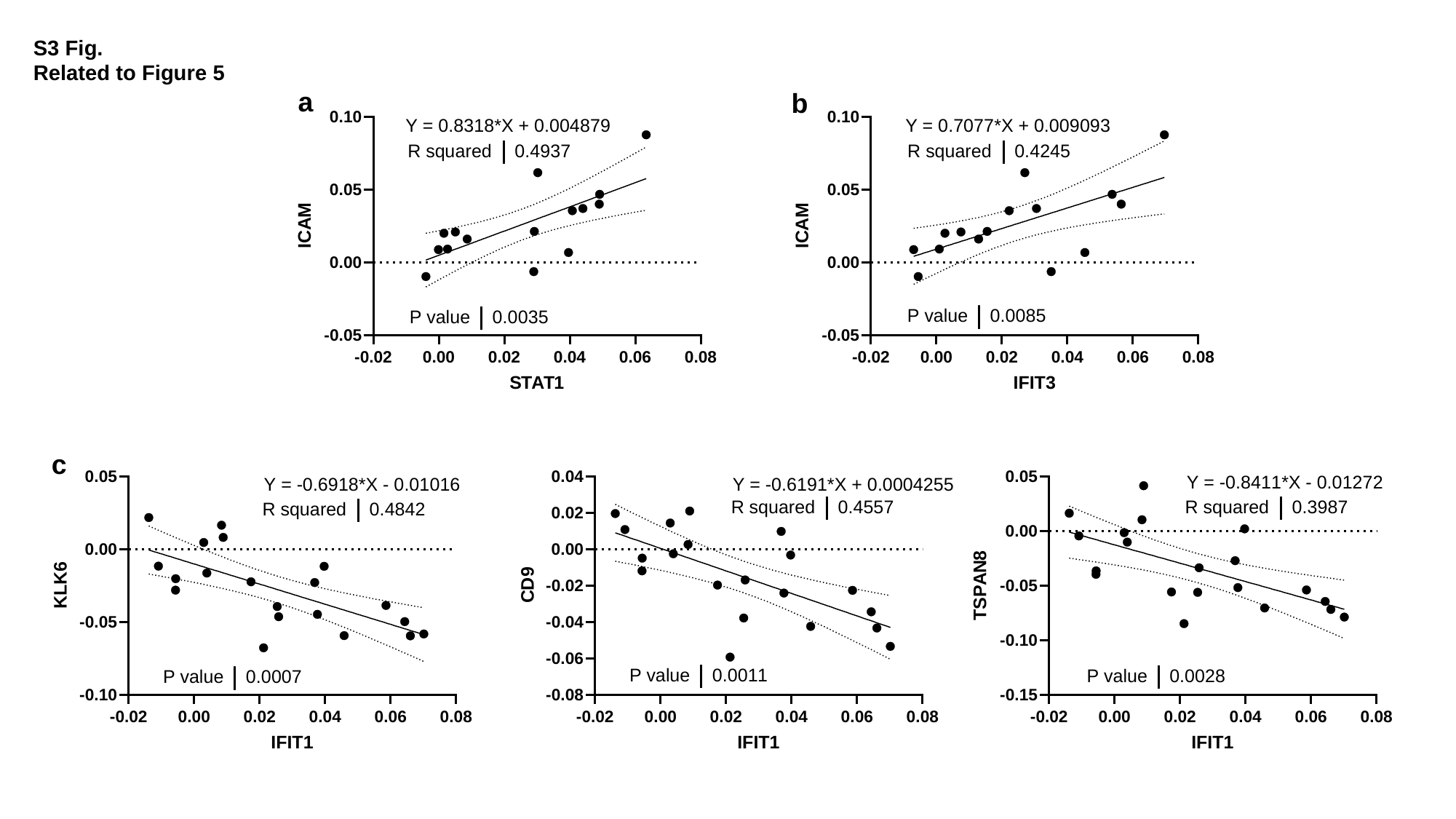

S3 Fig.
Related to Figure 5
a
b
c

Supplement: S3 Fig — ICAM protein expression was positively correlated with IFN-I signaling protein STAT1 (A) and IFIT3 (B), which was analyzed using linear regression. A similar analysis was performed between IFIT1 and a few down-regulated proteins essential for neuronal functions, including KLK6, CD9, and TSPAN8 (C). (PPTX) [file ppat.1013411.s003.pptx]

## Slide 1
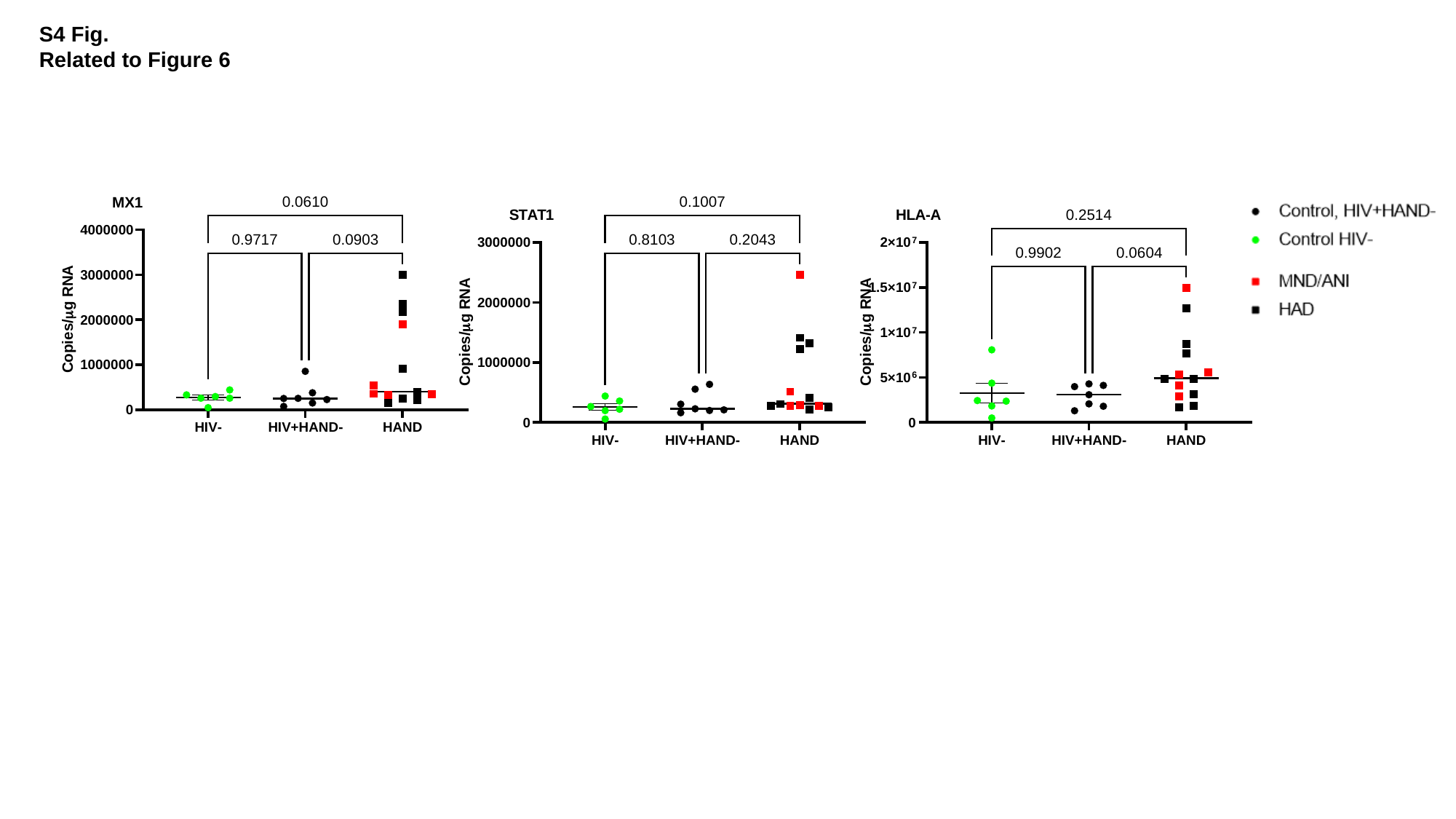

S4 Fig.
Related to Figure 6

Supplement: S4 Fig — MX1, STAT1, and HLA-A mRNA expression was analyzed by RT-qPCR in the HAND brains (n = 14), compared with HIV-negative brains (n = 6) and HIV+ but HAND- brains (n = 7). The p-value was calculated using Welch’s ANOVA comparison with controls. (PPTX) [file ppat.1013411.s004.pptx]

## Slide 1
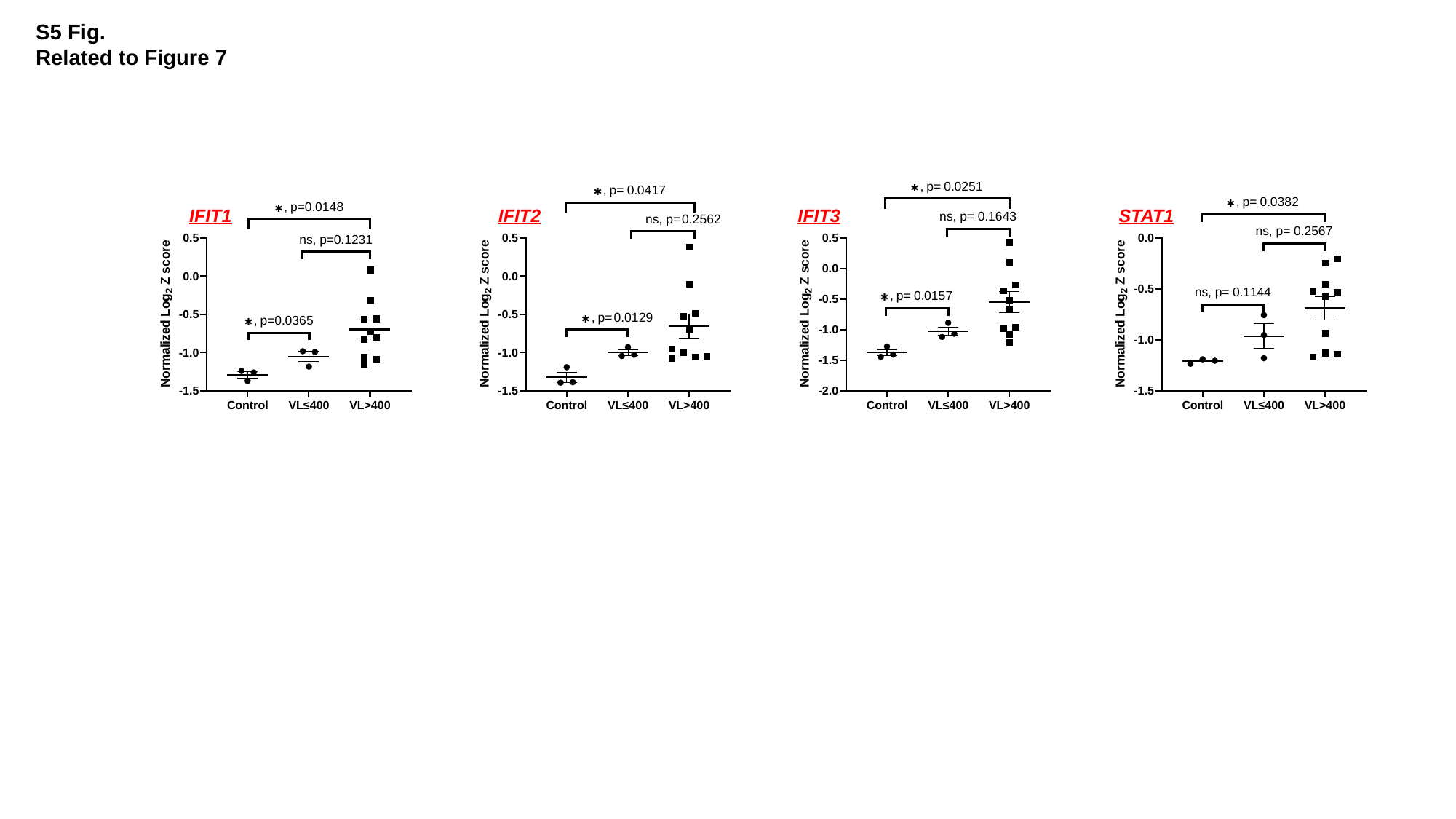

S5 Fig.
Related to Figure 7
IFIT1
IFIT2
IFIT3
STAT1

Supplement: S5 Fig — The expression of ISGs in HAND brains from PWH with VL > 400 copies/mL or VL < 400 copies/mL. Statistical differences were analyzed by Two-way ANOVA. (PPTX) [file ppat.1013411.s005.pptx]

## Slide 1
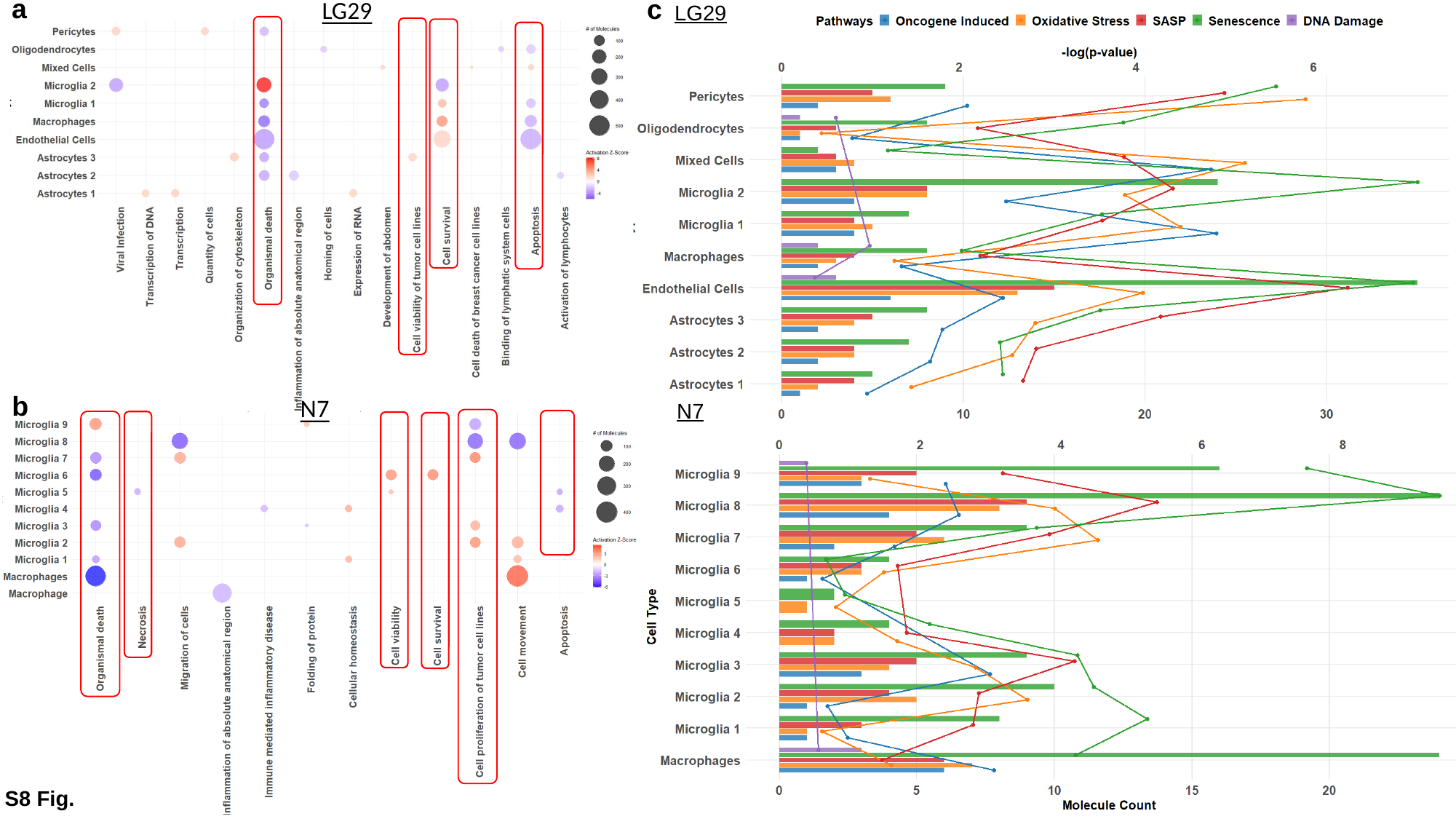

a
c
LG29
b
N7
S8 Fig.

Supplement: S8 Fig — Analysis of disease and function pathways uncovered altered cell death, survival, and proliferation signaling in CNS cells in LG29 (A) and N7 (B) on ART. (C) A study of proliferation genes in the subsets of myeloid cells on ART discovered unique subsets of MG that may acquire proliferation advantages critical for them to serve as stable viral reservoirs on long-term ART. (PPTX) [file ppat.1013411.s008.pptx]

## Slide 1
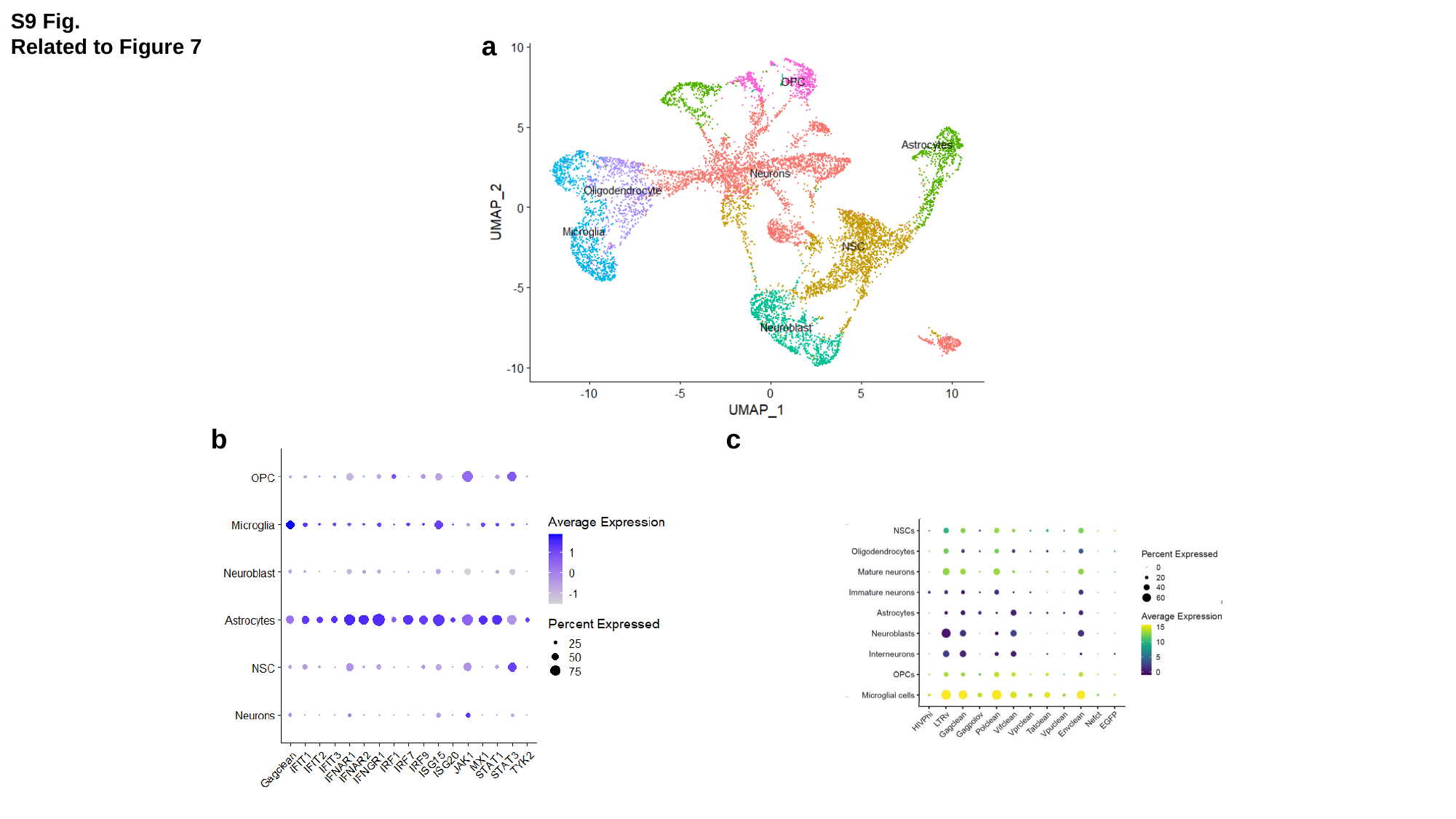

S9 Fig.
Related to Figure 7
a
b
c

Supplement: S9 Fig — MCO was established and infected by HIV for 7 days. Then, MCO was collected, and the single-cell suspension was prepared for scRNA-seq analyses of CNS cells (A), IFN-I activation (B), and HIV transcripts (C). (PPTX) [file ppat.1013411.s009.pptx]

## Slide 1
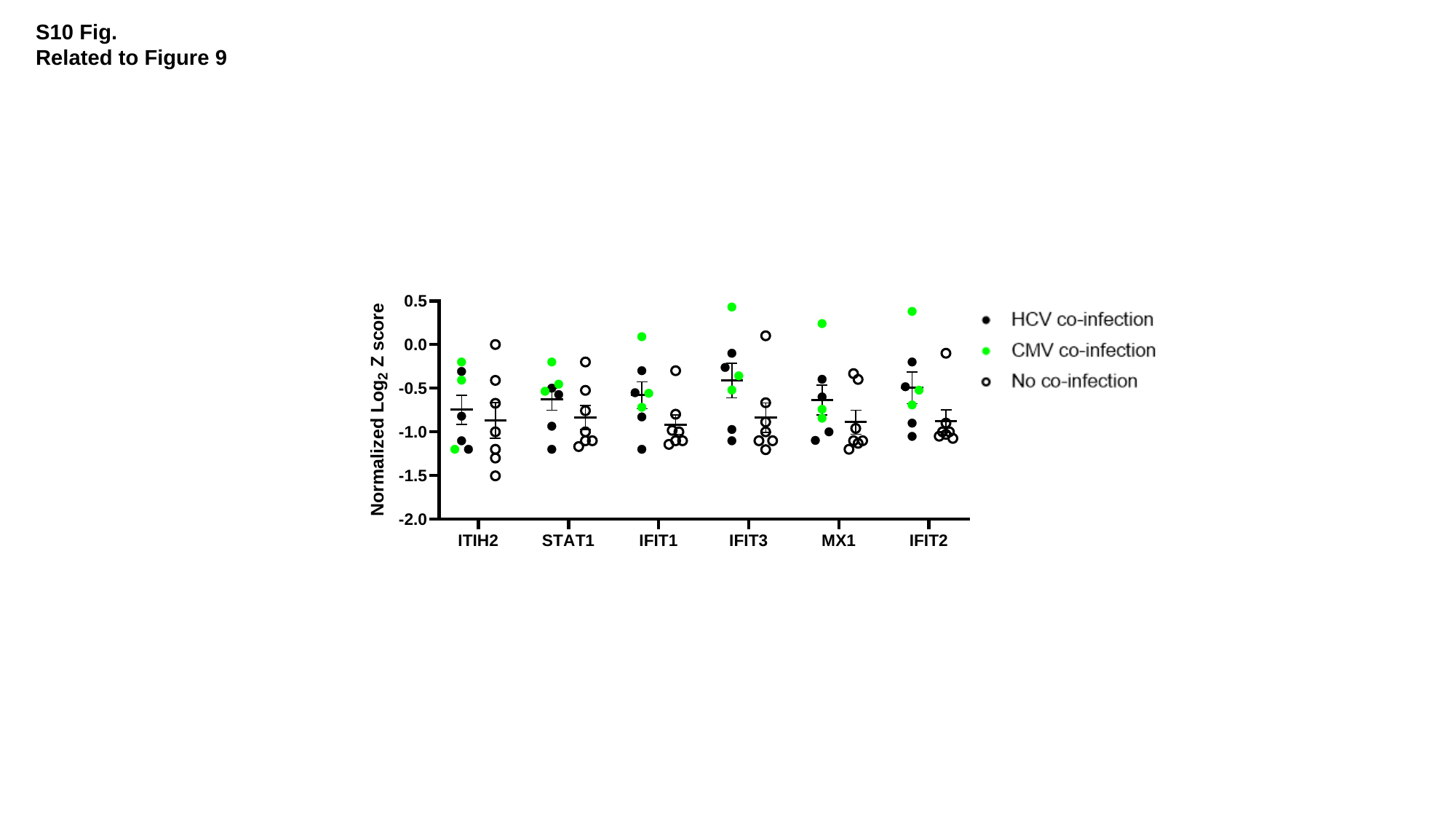

S10 Fig.
Related to Figure 9

Supplement: S10 Fig — The protein abundance Z-scores of IFN-I signaling genes in the WM of HAND brains were compared with the Z-scores of HAND brains co-infected with HCV or CMV, and a two-way ANOVA test was performed to calculate the p-value. (PPTX) [file ppat.1013411.s010.pptx]
