# Supplementary material for: Persistent type I interferon signaling within the brain of people with HIV on ART with cognitive impairment
Source: PLoS Pathog. 2025 Aug 20;21(8):e1013411. doi: 10.1371/journal.ppat.1013411 (PMC12367146; doi:10.1371/journal.ppat.1013411)
Supplement: S6 Fig — UMAP projection (A) and heatmap (B) showed the expression of the representative CNS cell biomarkers (for example, HEXB and TMEM119 for MG, GFAP for astrocytes, OLIG1 and OLIG2 for oligodendrocytes, CD68 and CD163 for macrophages, and ALCAM for pericytes). (PPTX) [file ppat.1013411.s006.pptx]

## Slide 1
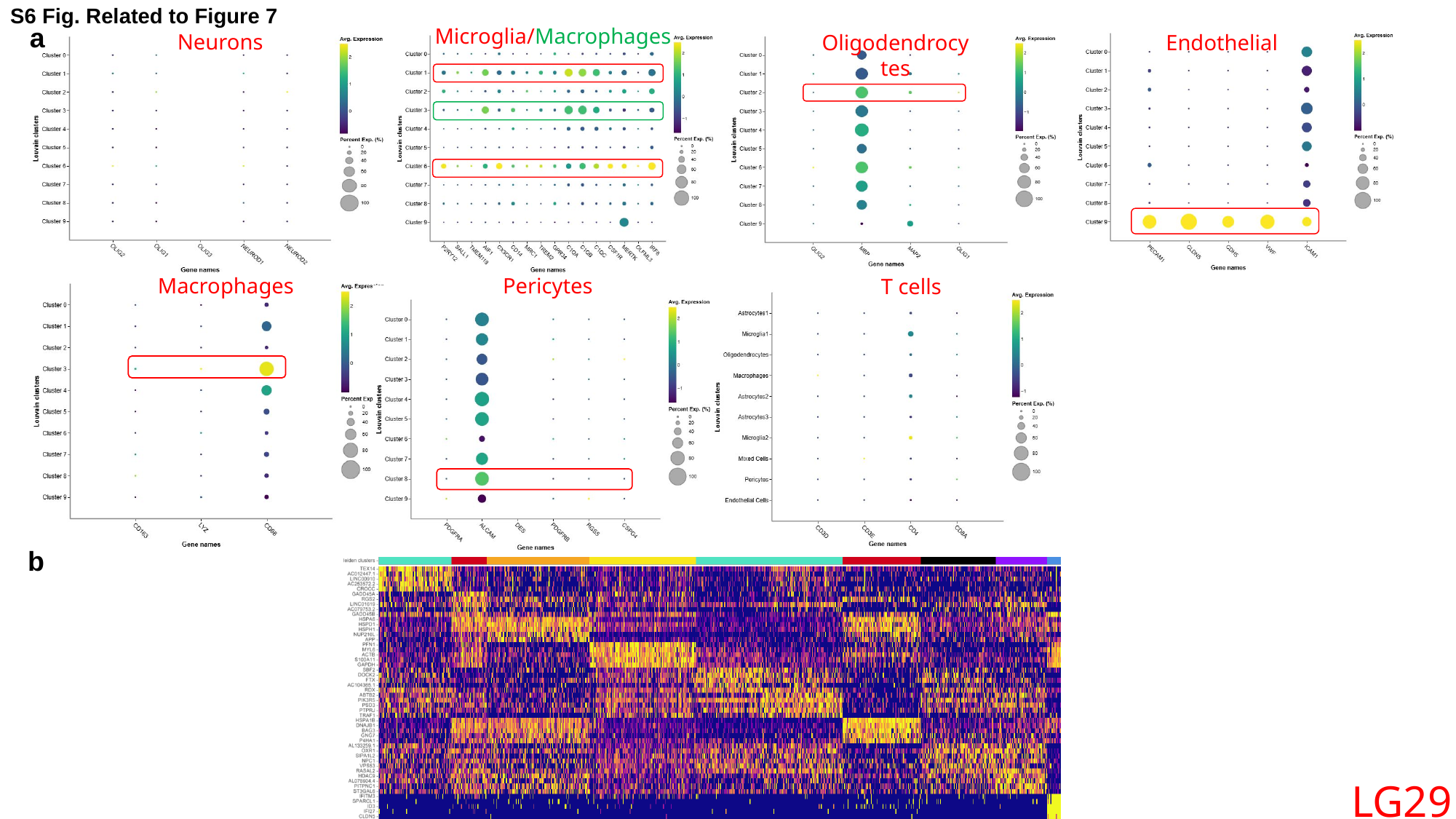

S6 Fig. Related to Figure 7
a
Microglia/Macrophages
Neurons
Endothelial
Oligodendrocytes
Macrophages
Pericytes
T cells
b
LG29
