# Supplementary material for: Persistent type I interferon signaling within the brain of people with HIV on ART with cognitive impairment
Source: PLoS Pathog. 2025 Aug 20;21(8):e1013411. doi: 10.1371/journal.ppat.1013411 (PMC12367146; doi:10.1371/journal.ppat.1013411)
Supplement: S7 Fig — Cluster analysis (A) and heatmap (B) showed the expression of representative biomarkers (HEXB and TMEM119 for MG; CD163 and CD68 for macrophages) of CNS cells. (PPTX) [file ppat.1013411.s007.pptx]

## Slide 1
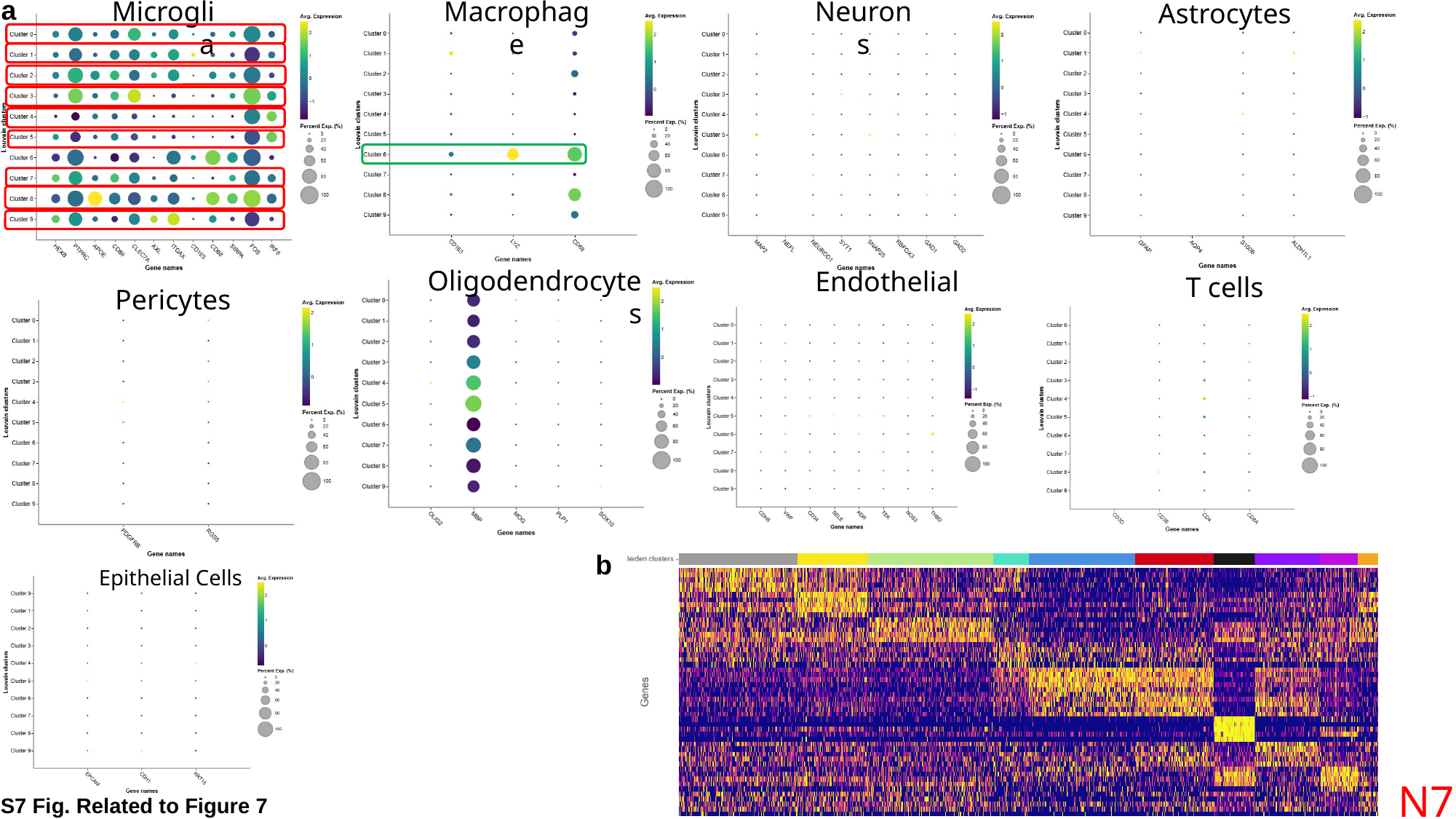

a
Microglia
Macrophage
Neurons
Astrocytes
Oligodendrocytes
Endothelial Cells
T cells
Pericytes
b
Epithelial Cells
N7
S7 Fig. Related to Figure 7
