# Supplementary material for: Persistent type I interferon signaling within the brain of people with HIV on ART with cognitive impairment
Source: PLoS Pathog. 2025 Aug 20;21(8):e1013411. doi: 10.1371/journal.ppat.1013411 (PMC12367146; doi:10.1371/journal.ppat.1013411)
Supplement: S1 Table — (PPTX) [file ppat.1013411.s011.pptx]

## Slide 1
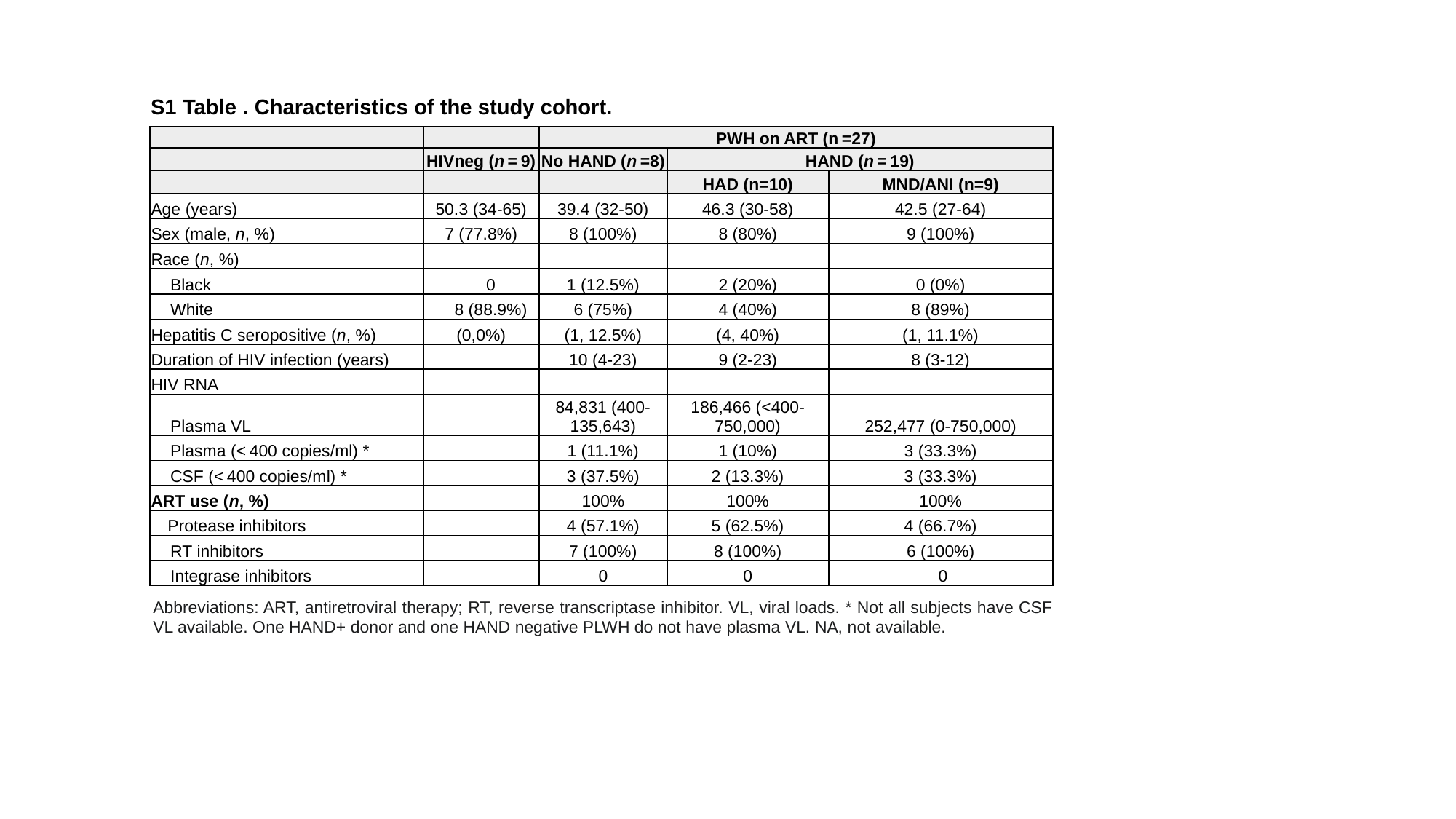

S1 Table . Characteristics of the study cohort.
| | | PWH on ART (n =27) | | |
| --- | --- | --- | --- | --- |
| | HIVneg (n = 9) | No HAND (n =8) | HAND (n = 19) | |
| | | | HAD (n=10) | MND/ANI (n=9) |
| Age (years) | 50.3 (34-65) | 39.4 (32-50) | 46.3 (30-58) | 42.5 (27-64) |
| Sex (male, n, %) | 7 (77.8%) | 8 (100%) | 8 (80%) | 9 (100%) |
| Race (n, %) | | | | |
| Black | 0 | 1 (12.5%) | 2 (20%) | 0 (0%) |
| White | 8 (88.9%) | 6 (75%) | 4 (40%) | 8 (89%) |
| Hepatitis C seropositive (n, %) | (0,0%) | (1, 12.5%) | (4, 40%) | (1, 11.1%) |
| Duration of HIV infection (years) | | 10 (4-23) | 9 (2-23) | 8 (3-12) |
| HIV RNA | | | | |
| Plasma VL | | 84,831 (400-135,643) | 186,466 (<400-750,000) | 252,477 (0-750,000) |
| Plasma (< 400 copies/ml) \* | | 1 (11.1%) | 1 (10%) | 3 (33.3%) |
| CSF (< 400 copies/ml) \* | | 3 (37.5%) | 2 (13.3%) | 3 (33.3%) |
| ART use (n, %) | | 100% | 100% | 100% |
| Protease inhibitors | | 4 (57.1%) | 5 (62.5%) | 4 (66.7%) |
| RT inhibitors | | 7 (100%) | 8 (100%) | 6 (100%) |
| Integrase inhibitors | | 0 | 0 | 0 |
Abbreviations: ART, antiretroviral therapy; RT, reverse transcriptase inhibitor. VL, viral loads. * Not all subjects have CSF VL available. One HAND+ donor and one HAND negative PLWH do not have plasma VL. NA, not available.
