# Supplementary material for: Persistent type I interferon signaling within the brain of people with HIV on ART with cognitive impairment
Source: PLoS Pathog. 2025 Aug 20;21(8):e1013411. doi: 10.1371/journal.ppat.1013411 (PMC12367146; doi:10.1371/journal.ppat.1013411)
Supplement: S2 Table — (PPTX) [file ppat.1013411.s012.pptx]

## Slide 1
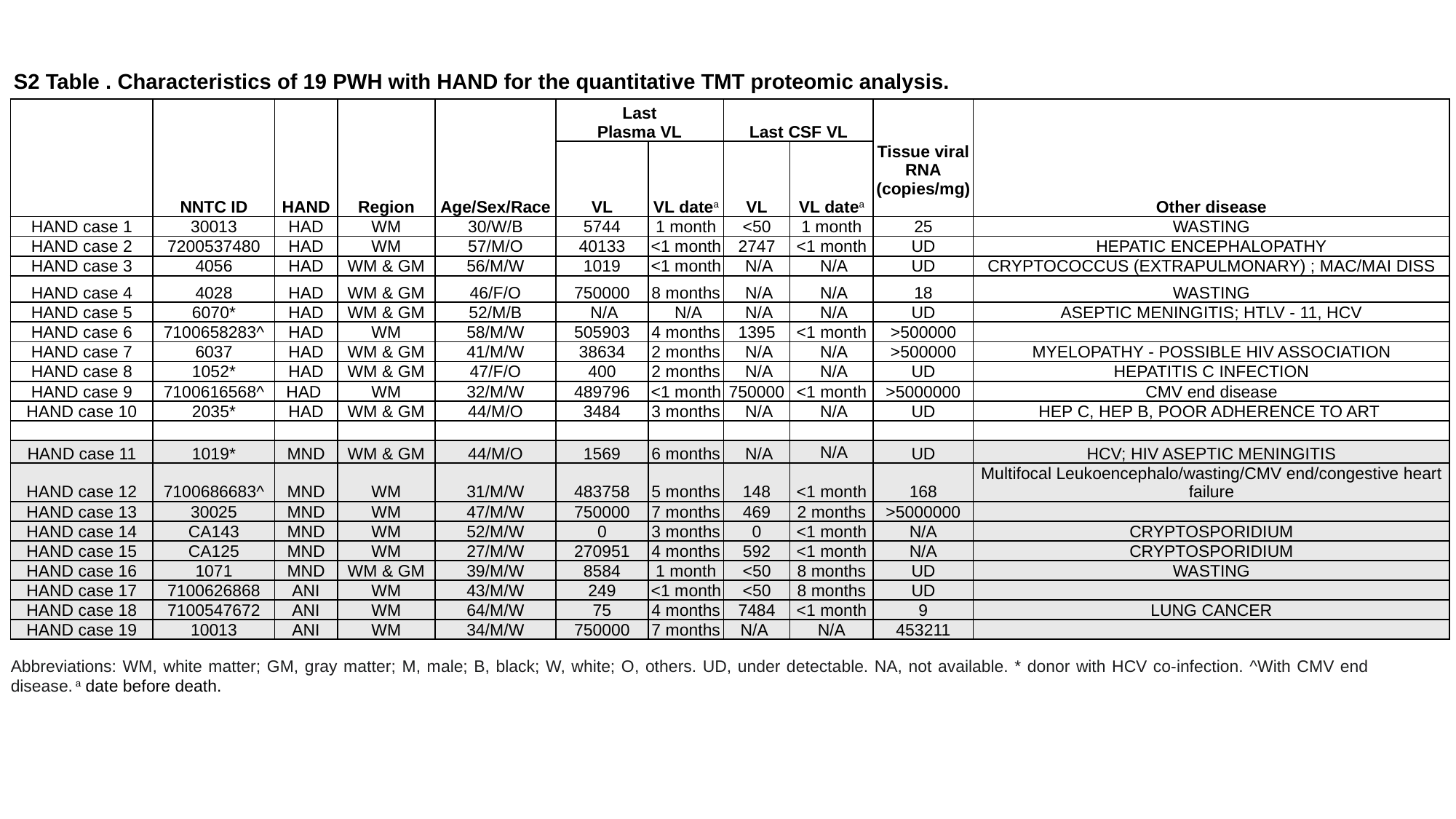

S2 Table . Characteristics of 19 PWH with HAND for the quantitative TMT proteomic analysis.
| | NNTC ID | HAND | Region | Age/Sex/Race | Last Plasma VL | | Last CSF VL | | Tissue viral RNA (copies/mg) | Other disease |
| --- | --- | --- | --- | --- | --- | --- | --- | --- | --- | --- |
| | | | | | VL | VL datea | VL | VL datea | | |
| HAND case 1 | 30013 | HAD | WM | 30/W/B | 5744 | 1 month | <50 | 1 month | 25 | WASTING |
| HAND case 2 | 7200537480 | HAD | WM | 57/M/O | 40133 | <1 month | 2747 | <1 month | UD | HEPATIC ENCEPHALOPATHY |
| HAND case 3 | 4056 | HAD | WM & GM | 56/M/W | 1019 | <1 month | N/A | N/A | UD | CRYPTOCOCCUS (EXTRAPULMONARY) ; MAC/MAI DISS |
| HAND case 4 | 4028 | HAD | WM & GM | 46/F/O | 750000 | 8 months | N/A | N/A | 18 | WASTING |
| HAND case 5 | 6070\* | HAD | WM & GM | 52/M/B | N/A | N/A | N/A | N/A | UD | ASEPTIC MENINGITIS; HTLV - 11, HCV |
| HAND case 6 | 7100658283^ | HAD | WM | 58/M/W | 505903 | 4 months | 1395 | <1 month | >500000 | |
| HAND case 7 | 6037 | HAD | WM & GM | 41/M/W | 38634 | 2 months | N/A | N/A | >500000 | MYELOPATHY - POSSIBLE HIV ASSOCIATION |
| HAND case 8 | 1052\* | HAD | WM & GM | 47/F/O | 400 | 2 months | N/A | N/A | UD | HEPATITIS C INFECTION |
| HAND case 9 | 7100616568^ | HAD | WM | 32/M/W | 489796 | <1 month | 750000 | <1 month | >5000000 | CMV end disease |
| HAND case 10 | 2035\* | HAD | WM & GM | 44/M/O | 3484 | 3 months | N/A | N/A | UD | HEP C, HEP B, POOR ADHERENCE TO ART |
| | | | | | | | | | | |
| HAND case 11 | 1019\* | MND | WM & GM | 44/M/O | 1569 | 6 months | N/A | N/A | UD | HCV; HIV ASEPTIC MENINGITIS |
| HAND case 12 | 7100686683^ | MND | WM | 31/M/W | 483758 | 5 months | 148 | <1 month | 168 | Multifocal Leukoencephalo/wasting/CMV end/congestive heart failure |
| HAND case 13 | 30025 | MND | WM | 47/M/W | 750000 | 7 months | 469 | 2 months | >5000000 | |
| HAND case 14 | CA143 | MND | WM | 52/M/W | 0 | 3 months | 0 | <1 month | N/A | CRYPTOSPORIDIUM |
| HAND case 15 | CA125 | MND | WM | 27/M/W | 270951 | 4 months | 592 | <1 month | N/A | CRYPTOSPORIDIUM |
| HAND case 16 | 1071 | MND | WM & GM | 39/M/W | 8584 | 1 month | <50 | 8 months | UD | WASTING |
| HAND case 17 | 7100626868 | ANI | WM | 43/M/W | 249 | <1 month | <50 | 8 months | UD | |
| HAND case 18 | 7100547672 | ANI | WM | 64/M/W | 75 | 4 months | 7484 | <1 month | 9 | LUNG CANCER |
| HAND case 19 | 10013 | ANI | WM | 34/M/W | 750000 | 7 months | N/A | N/A | 453211 | |
Abbreviations: WM, white matter; GM, gray matter; M, male; B, black; W, white; O, others. UD, under detectable. NA, not available. * donor with HCV co-infection. ^With CMV end disease. a date before death.
