# Supplementary material for: Persistent type I interferon signaling within the brain of people with HIV on ART with cognitive impairment
Source: PLoS Pathog. 2025 Aug 20;21(8):e1013411. doi: 10.1371/journal.ppat.1013411 (PMC12367146; doi:10.1371/journal.ppat.1013411)
Supplement: S3 Table — (PPTX) [file ppat.1013411.s013.pptx]

## Slide 1
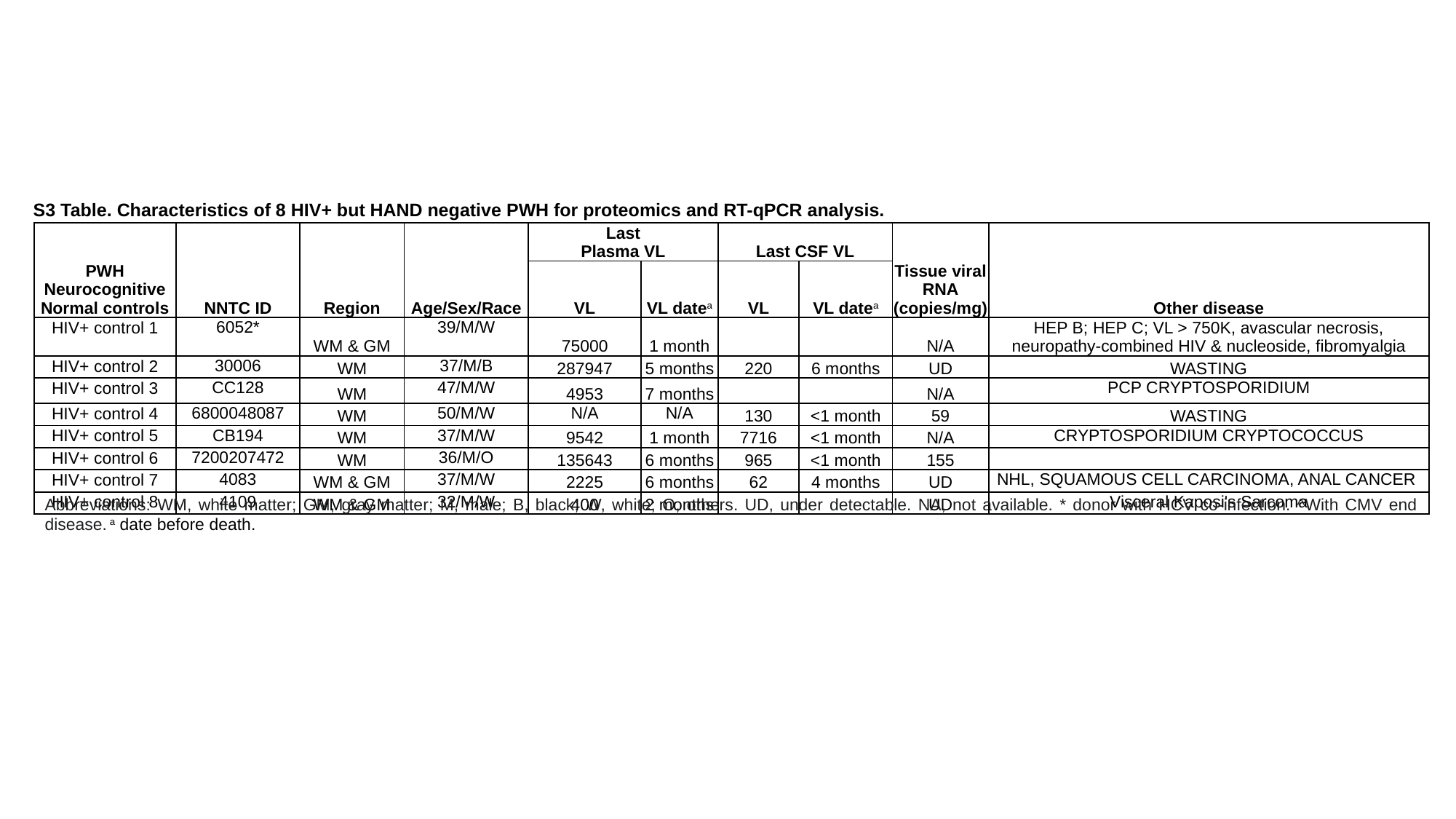

S3 Table. Characteristics of 8 HIV+ but HAND negative PWH for proteomics and RT-qPCR analysis.
| PWH Neurocognitive Normal controls | NNTC ID | Region | Age/Sex/Race | Last Plasma VL | | Last CSF VL | | Tissue viral RNA (copies/mg) | Other disease |
| --- | --- | --- | --- | --- | --- | --- | --- | --- | --- |
| | | | | VL | VL datea | VL | VL datea | | |
| HIV+ control 1 | 6052\* | WM & GM | 39/M/W | 75000 | 1 month | | | N/A | HEP B; HEP C; VL > 750K, avascular necrosis, neuropathy-combined HIV & nucleoside, fibromyalgia |
| HIV+ control 2 | 30006 | WM | 37/M/B | 287947 | 5 months | 220 | 6 months | UD | WASTING |
| HIV+ control 3 | CC128 | WM | 47/M/W | 4953 | 7 months | | | N/A | PCP CRYPTOSPORIDIUM |
| HIV+ control 4 | 6800048087 | WM | 50/M/W | N/A | N/A | 130 | <1 month | 59 | WASTING |
| HIV+ control 5 | CB194 | WM | 37/M/W | 9542 | 1 month | 7716 | <1 month | N/A | CRYPTOSPORIDIUM CRYPTOCOCCUS |
| HIV+ control 6 | 7200207472 | WM | 36/M/O | 135643 | 6 months | 965 | <1 month | 155 | |
| HIV+ control 7 | 4083 | WM & GM | 37/M/W | 2225 | 6 months | 62 | 4 months | UD | NHL, SQUAMOUS CELL CARCINOMA, ANAL CANCER |
| HIV+ control 8 | 4109 | WM & GM | 32/M/W | 400 | 2 months | | | UD | Visceral Kaposi's Sarcoma |
Abbreviations: WM, white matter; GM, gray matter; M, male; B, black; W, white; O, others. UD, under detectable. NA, not available. * donor with HCV co-infection. ^With CMV end disease. a date before death.
