# Supplementary material for: Persistent type I interferon signaling within the brain of people with HIV on ART with cognitive impairment
Source: PLoS Pathog. 2025 Aug 20;21(8):e1013411. doi: 10.1371/journal.ppat.1013411 (PMC12367146; doi:10.1371/journal.ppat.1013411)
Supplement: S4 Table — (PPTX) [file ppat.1013411.s014.pptx]

## Slide 1
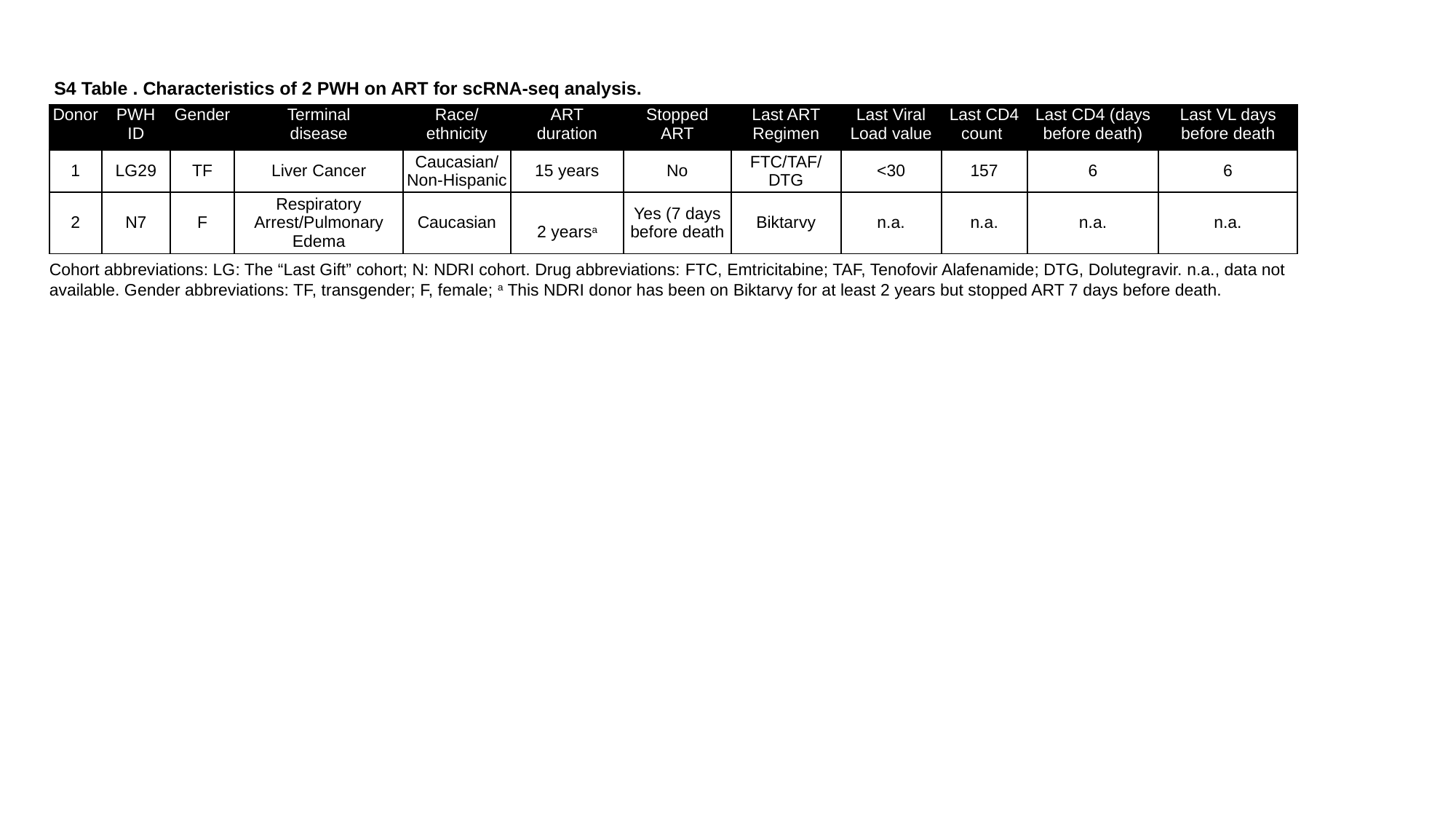

S4 Table . Characteristics of 2 PWH on ART for scRNA-seq analysis.
| Donor | PWH ID | Gender | Terminal disease | Race/ ethnicity | ART duration | Stopped ART | Last ART Regimen | Last Viral Load value | Last CD4 count | Last CD4 (days before death) | Last VL days before death |
| --- | --- | --- | --- | --- | --- | --- | --- | --- | --- | --- | --- |
| 1 | LG29 | TF | Liver Cancer | Caucasian/ Non-Hispanic | 15 years | No | FTC/TAF/ DTG | <30 | 157 | 6 | 6 |
| 2 | N7 | F | Respiratory Arrest/Pulmonary Edema | Caucasian | 2 yearsa | Yes (7 days before death | Biktarvy | n.a. | n.a. | n.a. | n.a. |
Cohort abbreviations: LG: The “Last Gift” cohort; N: NDRI cohort. Drug abbreviations: FTC, Emtricitabine; TAF, Tenofovir Alafenamide; DTG, Dolutegravir. n.a., data not available. Gender abbreviations: TF, transgender; F, female; a This NDRI donor has been on Biktarvy for at least 2 years but stopped ART 7 days before death.
