# Supplementary material for: Persistent type I interferon signaling within the brain of people with HIV on ART with cognitive impairment
Source: PLoS Pathog. 2025 Aug 20;21(8):e1013411. doi: 10.1371/journal.ppat.1013411 (PMC12367146; doi:10.1371/journal.ppat.1013411)
Supplement: S5 Table — (PPTX) [file ppat.1013411.s015.pptx]

## Slide 1
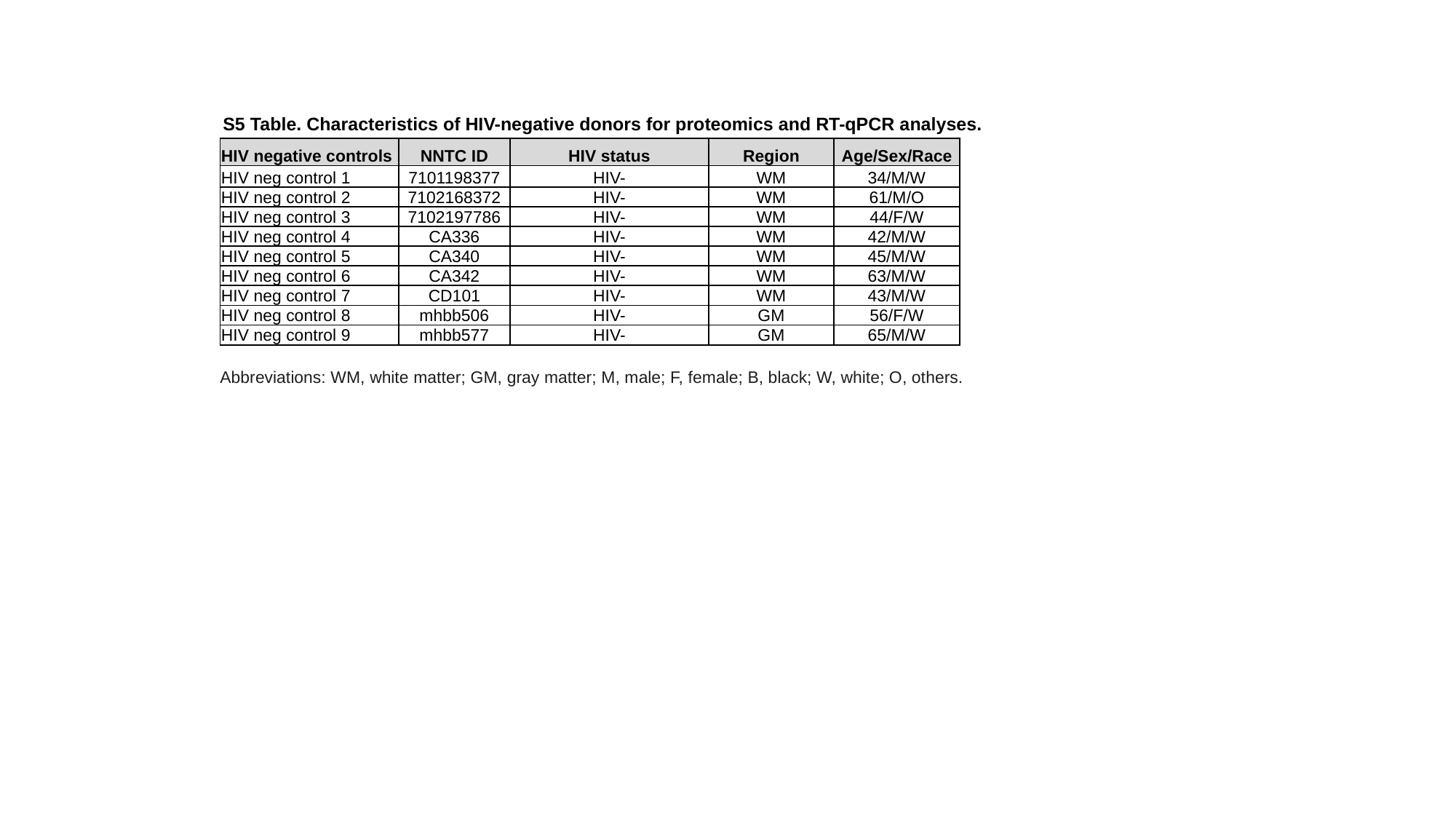

S5 Table. Characteristics of HIV-negative donors for proteomics and RT-qPCR analyses.
| HIV negative controls | NNTC ID | HIV status | Region | Age/Sex/Race |
| --- | --- | --- | --- | --- |
| HIV neg control 1 | 7101198377 | HIV- | WM | 34/M/W |
| HIV neg control 2 | 7102168372 | HIV- | WM | 61/M/O |
| HIV neg control 3 | 7102197786 | HIV- | WM | 44/F/W |
| HIV neg control 4 | CA336 | HIV- | WM | 42/M/W |
| HIV neg control 5 | CA340 | HIV- | WM | 45/M/W |
| HIV neg control 6 | CA342 | HIV- | WM | 63/M/W |
| HIV neg control 7 | CD101 | HIV- | WM | 43/M/W |
| HIV neg control 8 | mhbb506 | HIV- | GM | 56/F/W |
| HIV neg control 9 | mhbb577 | HIV- | GM | 65/M/W |
Abbreviations: WM, white matter; GM, gray matter; M, male; F, female; B, black; W, white; O, others.
