# Supplementary material for: Persistent type I interferon signaling within the brain of people with HIV on ART with cognitive impairment
Source: PLoS Pathog. 2025 Aug 20;21(8):e1013411. doi: 10.1371/journal.ppat.1013411 (PMC12367146; doi:10.1371/journal.ppat.1013411)
Supplement: S6 Table — (PPTX) [file ppat.1013411.s016.pptx]

## Slide 1
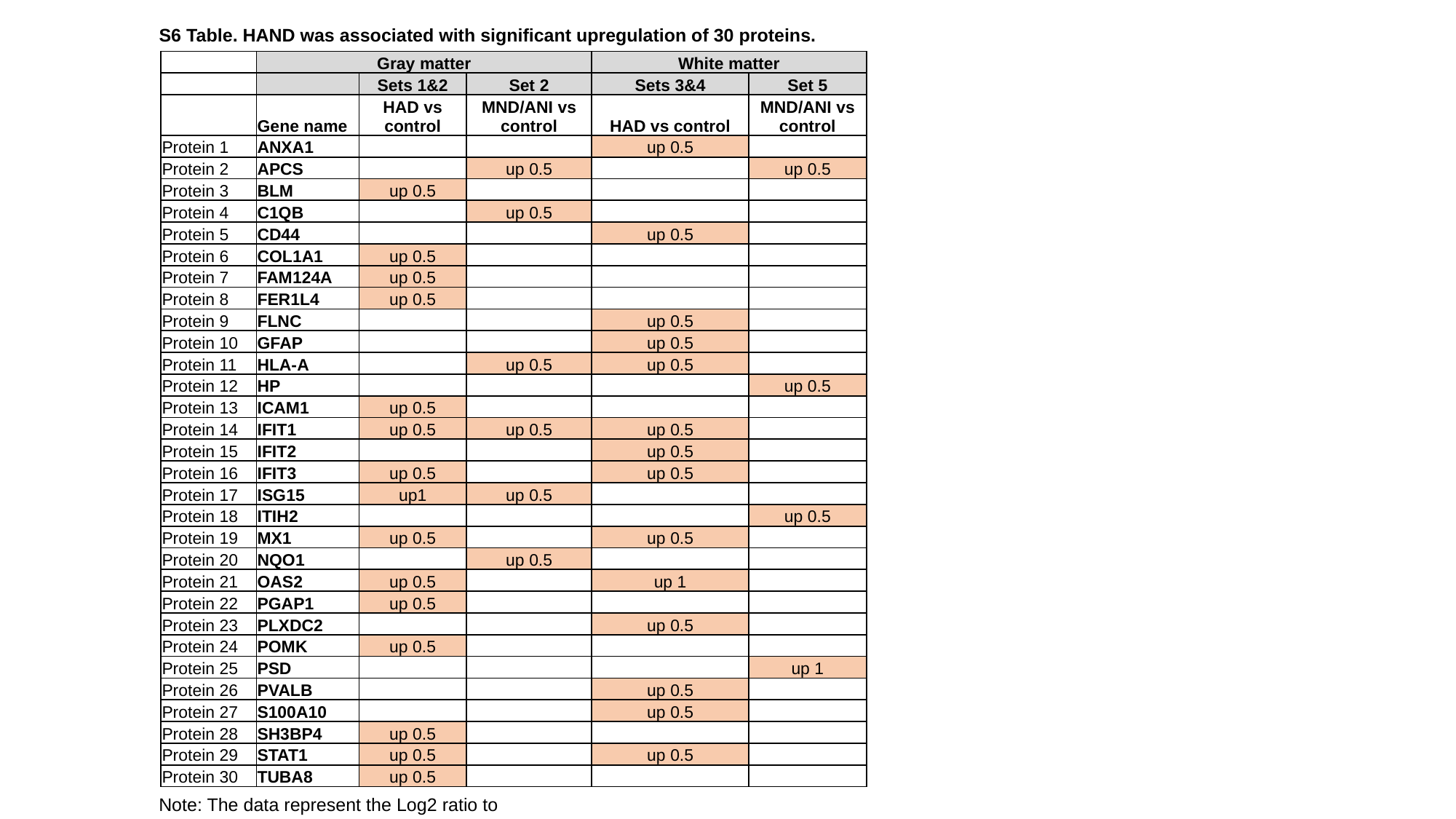

S6 Table. HAND was associated with significant upregulation of 30 proteins.
| | Gray matter | | | White matter | |
| --- | --- | --- | --- | --- | --- |
| | | Sets 1&2 | Set 2 | Sets 3&4 | Set 5 |
| | Gene name | HAD vs control | MND/ANI vs control | HAD vs control | MND/ANI vs control |
| Protein 1 | ANXA1 | | | up 0.5 | |
| Protein 2 | APCS | | up 0.5 | | up 0.5 |
| Protein 3 | BLM | up 0.5 | | | |
| Protein 4 | C1QB | | up 0.5 | | |
| Protein 5 | CD44 | | | up 0.5 | |
| Protein 6 | COL1A1 | up 0.5 | | | |
| Protein 7 | FAM124A | up 0.5 | | | |
| Protein 8 | FER1L4 | up 0.5 | | | |
| Protein 9 | FLNC | | | up 0.5 | |
| Protein 10 | GFAP | | | up 0.5 | |
| Protein 11 | HLA-A | | up 0.5 | up 0.5 | |
| Protein 12 | HP | | | | up 0.5 |
| Protein 13 | ICAM1 | up 0.5 | | | |
| Protein 14 | IFIT1 | up 0.5 | up 0.5 | up 0.5 | |
| Protein 15 | IFIT2 | | | up 0.5 | |
| Protein 16 | IFIT3 | up 0.5 | | up 0.5 | |
| Protein 17 | ISG15 | up1 | up 0.5 | | |
| Protein 18 | ITIH2 | | | | up 0.5 |
| Protein 19 | MX1 | up 0.5 | | up 0.5 | |
| Protein 20 | NQO1 | | up 0.5 | | |
| Protein 21 | OAS2 | up 0.5 | | up 1 | |
| Protein 22 | PGAP1 | up 0.5 | | | |
| Protein 23 | PLXDC2 | | | up 0.5 | |
| Protein 24 | POMK | up 0.5 | | | |
| Protein 25 | PSD | | | | up 1 |
| Protein 26 | PVALB | | | up 0.5 | |
| Protein 27 | S100A10 | | | up 0.5 | |
| Protein 28 | SH3BP4 | up 0.5 | | | |
| Protein 29 | STAT1 | up 0.5 | | up 0.5 | |
| Protein 30 | TUBA8 | up 0.5 | | | |
Note: The data represent the Log2 ratio to control.
