# Supplementary material for: Persistent type I interferon signaling within the brain of people with HIV on ART with cognitive impairment
Source: PLoS Pathog. 2025 Aug 20;21(8):e1013411. doi: 10.1371/journal.ppat.1013411 (PMC12367146; doi:10.1371/journal.ppat.1013411)
Supplement: S7 Table — (PPTX) [file ppat.1013411.s017.pptx]

## Slide 1
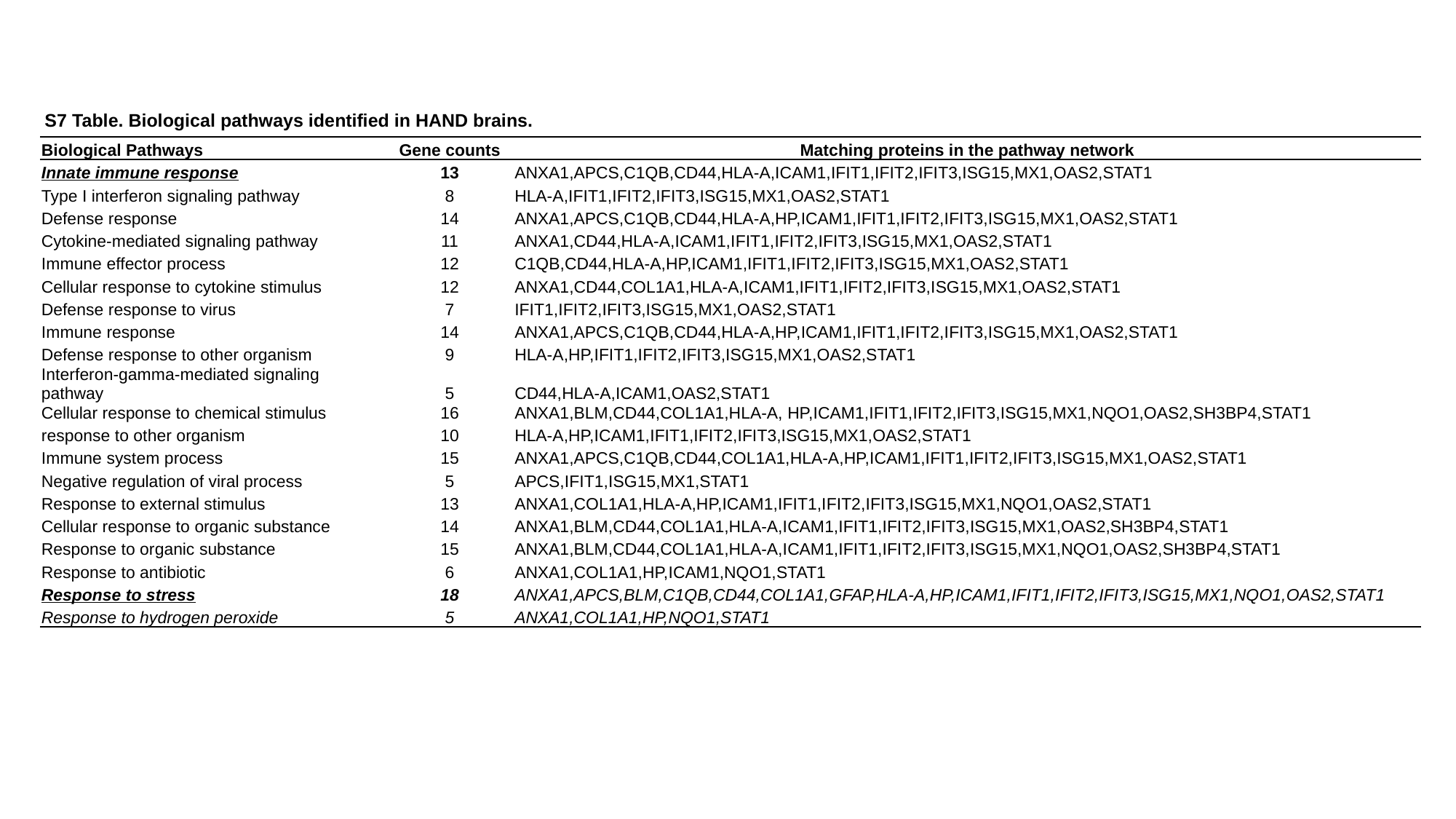

S7 Table. Biological pathways identified in HAND brains.
| Biological Pathways | Gene counts | Matching proteins in the pathway network |
| --- | --- | --- |
| Innate immune response | 13 | ANXA1,APCS,C1QB,CD44,HLA-A,ICAM1,IFIT1,IFIT2,IFIT3,ISG15,MX1,OAS2,STAT1 |
| Type I interferon signaling pathway | 8 | HLA-A,IFIT1,IFIT2,IFIT3,ISG15,MX1,OAS2,STAT1 |
| Defense response | 14 | ANXA1,APCS,C1QB,CD44,HLA-A,HP,ICAM1,IFIT1,IFIT2,IFIT3,ISG15,MX1,OAS2,STAT1 |
| Cytokine-mediated signaling pathway | 11 | ANXA1,CD44,HLA-A,ICAM1,IFIT1,IFIT2,IFIT3,ISG15,MX1,OAS2,STAT1 |
| Immune effector process | 12 | C1QB,CD44,HLA-A,HP,ICAM1,IFIT1,IFIT2,IFIT3,ISG15,MX1,OAS2,STAT1 |
| Cellular response to cytokine stimulus | 12 | ANXA1,CD44,COL1A1,HLA-A,ICAM1,IFIT1,IFIT2,IFIT3,ISG15,MX1,OAS2,STAT1 |
| Defense response to virus | 7 | IFIT1,IFIT2,IFIT3,ISG15,MX1,OAS2,STAT1 |
| Immune response | 14 | ANXA1,APCS,C1QB,CD44,HLA-A,HP,ICAM1,IFIT1,IFIT2,IFIT3,ISG15,MX1,OAS2,STAT1 |
| Defense response to other organism | 9 | HLA-A,HP,IFIT1,IFIT2,IFIT3,ISG15,MX1,OAS2,STAT1 |
| Interferon-gamma-mediated signaling pathway | 5 | CD44,HLA-A,ICAM1,OAS2,STAT1 |
| Cellular response to chemical stimulus | 16 | ANXA1,BLM,CD44,COL1A1,HLA-A, HP,ICAM1,IFIT1,IFIT2,IFIT3,ISG15,MX1,NQO1,OAS2,SH3BP4,STAT1 |
| response to other organism | 10 | HLA-A,HP,ICAM1,IFIT1,IFIT2,IFIT3,ISG15,MX1,OAS2,STAT1 |
| Immune system process | 15 | ANXA1,APCS,C1QB,CD44,COL1A1,HLA-A,HP,ICAM1,IFIT1,IFIT2,IFIT3,ISG15,MX1,OAS2,STAT1 |
| Negative regulation of viral process | 5 | APCS,IFIT1,ISG15,MX1,STAT1 |
| Response to external stimulus | 13 | ANXA1,COL1A1,HLA-A,HP,ICAM1,IFIT1,IFIT2,IFIT3,ISG15,MX1,NQO1,OAS2,STAT1 |
| Cellular response to organic substance | 14 | ANXA1,BLM,CD44,COL1A1,HLA-A,ICAM1,IFIT1,IFIT2,IFIT3,ISG15,MX1,OAS2,SH3BP4,STAT1 |
| Response to organic substance | 15 | ANXA1,BLM,CD44,COL1A1,HLA-A,ICAM1,IFIT1,IFIT2,IFIT3,ISG15,MX1,NQO1,OAS2,SH3BP4,STAT1 |
| Response to antibiotic | 6 | ANXA1,COL1A1,HP,ICAM1,NQO1,STAT1 |
| Response to stress | 18 | ANXA1,APCS,BLM,C1QB,CD44,COL1A1,GFAP,HLA-A,HP,ICAM1,IFIT1,IFIT2,IFIT3,ISG15,MX1,NQO1,OAS2,STAT1 |
| Response to hydrogen peroxide | 5 | ANXA1,COL1A1,HP,NQO1,STAT1 |
