# Supplementary material for: Persistent type I interferon signaling within the brain of people with HIV on ART with cognitive impairment
Source: PLoS Pathog. 2025 Aug 20;21(8):e1013411. doi: 10.1371/journal.ppat.1013411 (PMC12367146; doi:10.1371/journal.ppat.1013411)
Supplement: S8 Table — (PPTX) [file ppat.1013411.s018.pptx]

## Slide 1
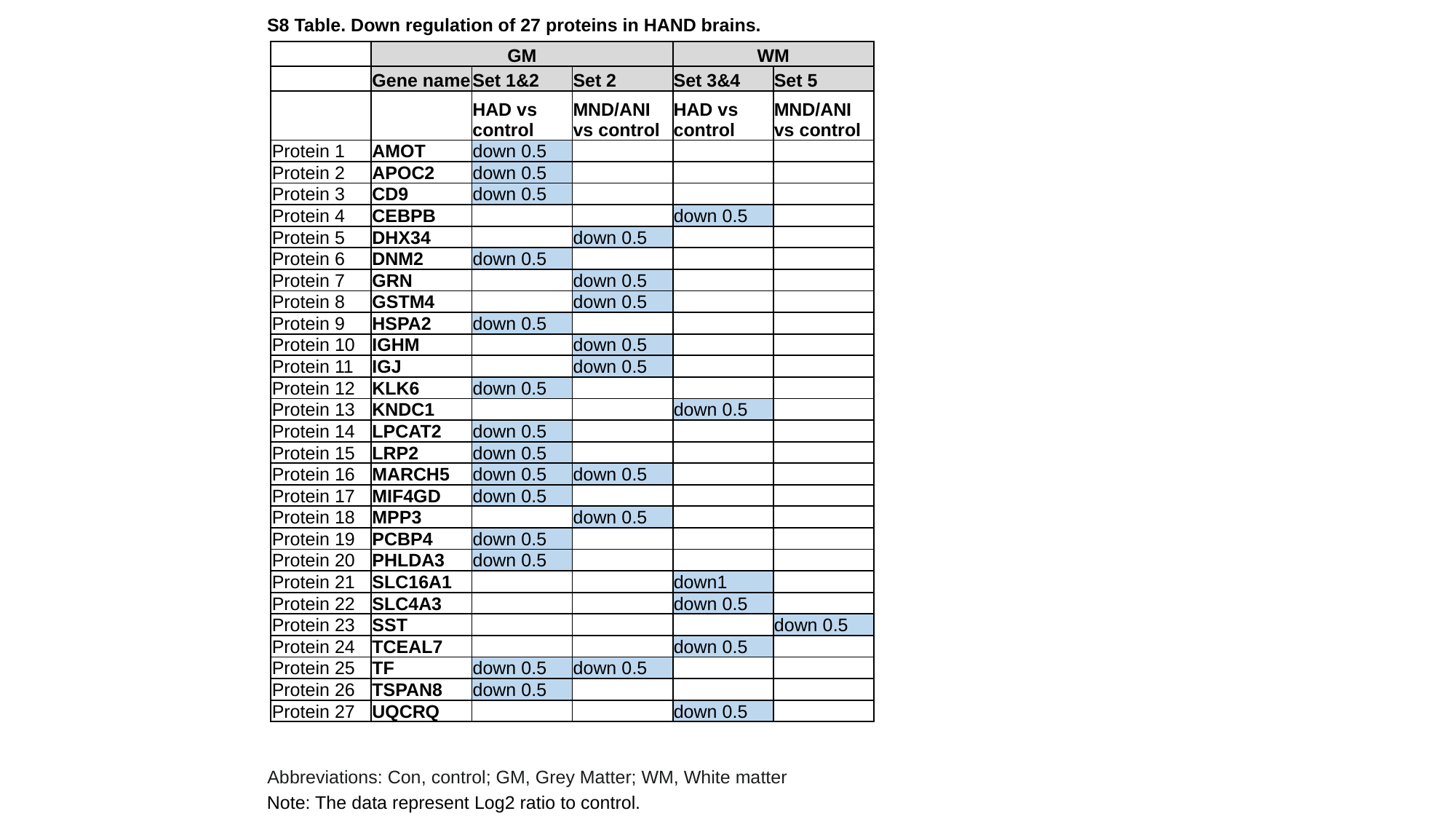

S8 Table. Down regulation of 27 proteins in HAND brains.
| | GM | | | WM | |
| --- | --- | --- | --- | --- | --- |
| | Gene name | Set 1&2 | Set 2 | Set 3&4 | Set 5 |
| | | HAD vs control | MND/ANI vs control | HAD vs control | MND/ANI vs control |
| Protein 1 | AMOT | down 0.5 | | | |
| Protein 2 | APOC2 | down 0.5 | | | |
| Protein 3 | CD9 | down 0.5 | | | |
| Protein 4 | CEBPB | | | down 0.5 | |
| Protein 5 | DHX34 | | down 0.5 | | |
| Protein 6 | DNM2 | down 0.5 | | | |
| Protein 7 | GRN | | down 0.5 | | |
| Protein 8 | GSTM4 | | down 0.5 | | |
| Protein 9 | HSPA2 | down 0.5 | | | |
| Protein 10 | IGHM | | down 0.5 | | |
| Protein 11 | IGJ | | down 0.5 | | |
| Protein 12 | KLK6 | down 0.5 | | | |
| Protein 13 | KNDC1 | | | down 0.5 | |
| Protein 14 | LPCAT2 | down 0.5 | | | |
| Protein 15 | LRP2 | down 0.5 | | | |
| Protein 16 | MARCH5 | down 0.5 | down 0.5 | | |
| Protein 17 | MIF4GD | down 0.5 | | | |
| Protein 18 | MPP3 | | down 0.5 | | |
| Protein 19 | PCBP4 | down 0.5 | | | |
| Protein 20 | PHLDA3 | down 0.5 | | | |
| Protein 21 | SLC16A1 | | | down1 | |
| Protein 22 | SLC4A3 | | | down 0.5 | |
| Protein 23 | SST | | | | down 0.5 |
| Protein 24 | TCEAL7 | | | down 0.5 | |
| Protein 25 | TF | down 0.5 | down 0.5 | | |
| Protein 26 | TSPAN8 | down 0.5 | | | |
| Protein 27 | UQCRQ | | | down 0.5 | |
Abbreviations: Con, control; GM, Grey Matter; WM, White matter
Note: The data represent Log2 ratio to control.
