# Supplementary material for: Persistent type I interferon signaling within the brain of people with HIV on ART with cognitive impairment
Source: PLoS Pathog. 2025 Aug 20;21(8):e1013411. doi: 10.1371/journal.ppat.1013411 (PMC12367146; doi:10.1371/journal.ppat.1013411)
Supplement: S9 Table — (PPTX) [file ppat.1013411.s019.pptx]

## Slide 1
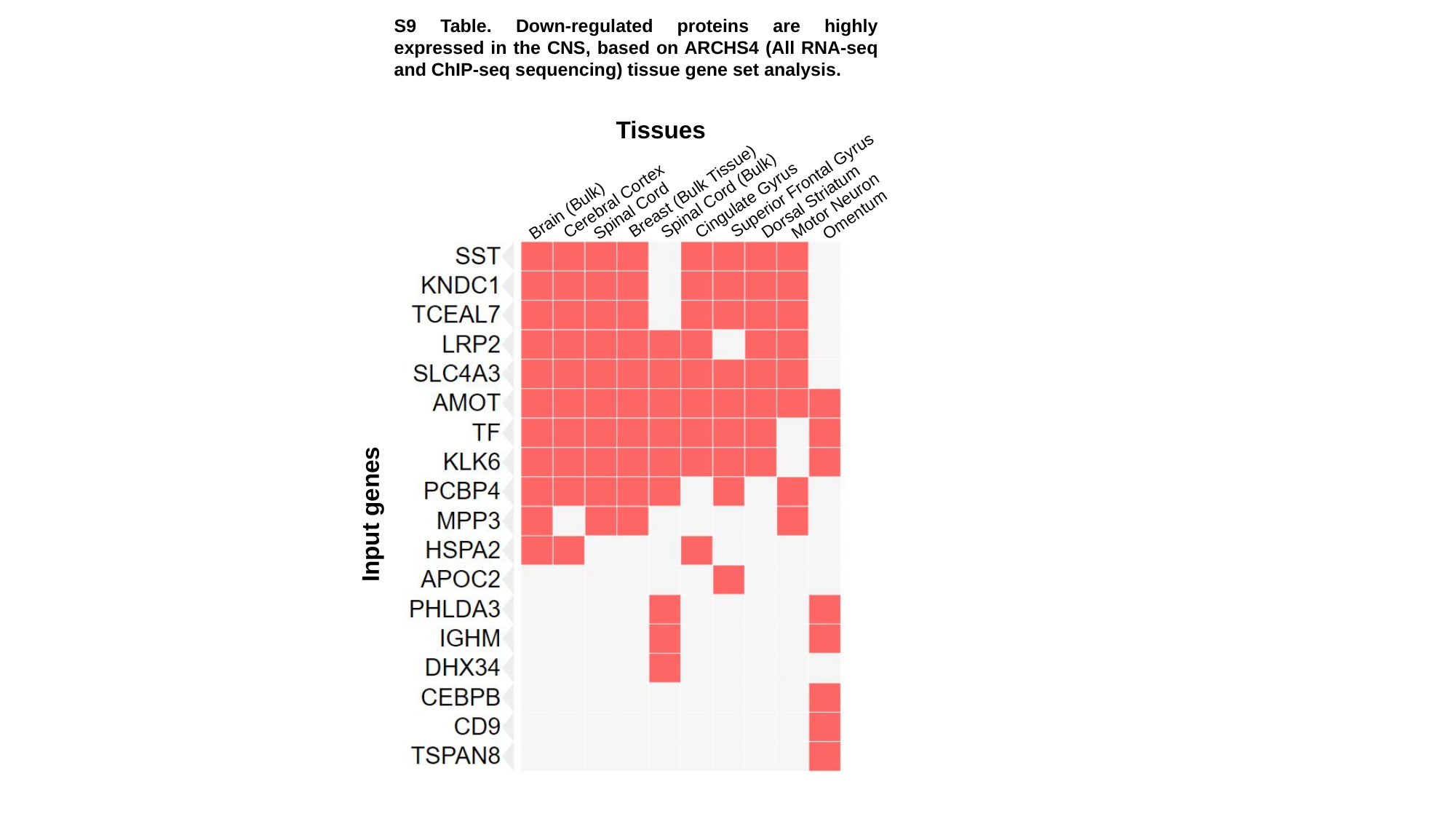

S9 Table. Down-regulated proteins are highly expressed in the CNS, based on ARCHS4 (All RNA-seq and ChIP-seq sequencing) tissue gene set analysis.
Tissues
Superior Frontal Gyrus
Breast (Bulk Tissue)
Spinal Cord (Bulk)
Cingulate Gyrus
Cerebral Cortex
Dorsal Striatum
Motor Neuron
Brain (Bulk)
Spinal Cord
Omentum
Input genes
