# Supplementary material for: Persistent type I interferon signaling within the brain of people with HIV on ART with cognitive impairment
Source: PLoS Pathog. 2025 Aug 20;21(8):e1013411. doi: 10.1371/journal.ppat.1013411 (PMC12367146; doi:10.1371/journal.ppat.1013411)
Supplement: S10 Table — (PPTX) [file ppat.1013411.s020.pptx]

## Slide 1
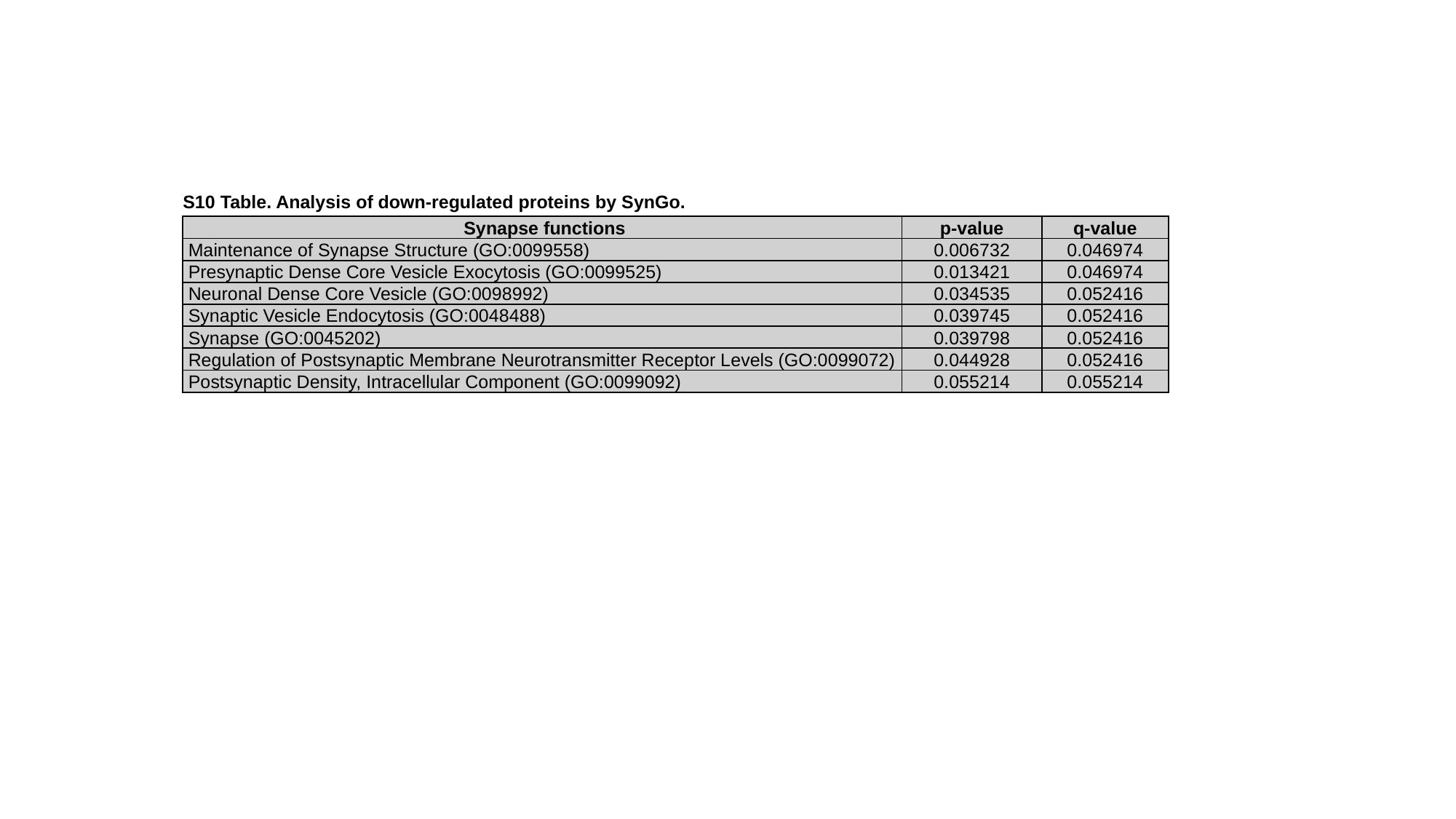

S10 Table. Analysis of down-regulated proteins by SynGo.
| Synapse functions | p-value | q-value |
| --- | --- | --- |
| Maintenance of Synapse Structure (GO:0099558) | 0.006732 | 0.046974 |
| Presynaptic Dense Core Vesicle Exocytosis (GO:0099525) | 0.013421 | 0.046974 |
| Neuronal Dense Core Vesicle (GO:0098992) | 0.034535 | 0.052416 |
| Synaptic Vesicle Endocytosis (GO:0048488) | 0.039745 | 0.052416 |
| Synapse (GO:0045202) | 0.039798 | 0.052416 |
| Regulation of Postsynaptic Membrane Neurotransmitter Receptor Levels (GO:0099072) | 0.044928 | 0.052416 |
| Postsynaptic Density, Intracellular Component (GO:0099092) | 0.055214 | 0.055214 |
