# Supplementary material for: Persistent type I interferon signaling within the brain of people with HIV on ART with cognitive impairment
Source: PLoS Pathog. 2025 Aug 20;21(8):e1013411. doi: 10.1371/journal.ppat.1013411 (PMC12367146; doi:10.1371/journal.ppat.1013411)
Supplement: S11 Table — (PPTX) [file ppat.1013411.s021.pptx]

## Slide 1
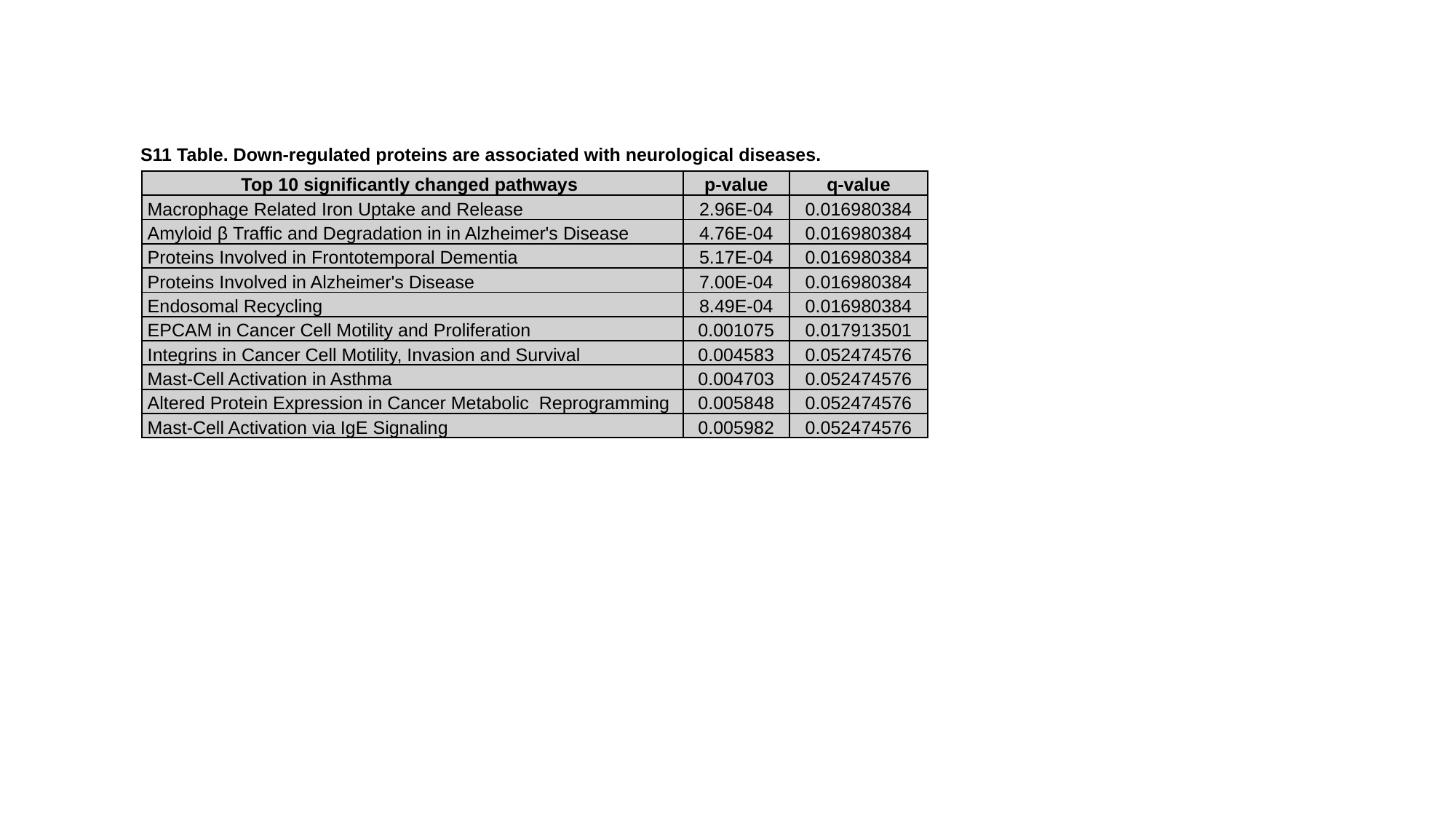

S11 Table. Down-regulated proteins are associated with neurological diseases.
| Top 10 significantly changed pathways | p-value | q-value |
| --- | --- | --- |
| Macrophage Related Iron Uptake and Release | 2.96E-04 | 0.016980384 |
| Amyloid β Traffic and Degradation in in Alzheimer's Disease | 4.76E-04 | 0.016980384 |
| Proteins Involved in Frontotemporal Dementia | 5.17E-04 | 0.016980384 |
| Proteins Involved in Alzheimer's Disease | 7.00E-04 | 0.016980384 |
| Endosomal Recycling | 8.49E-04 | 0.016980384 |
| EPCAM in Cancer Cell Motility and Proliferation | 0.001075 | 0.017913501 |
| Integrins in Cancer Cell Motility, Invasion and Survival | 0.004583 | 0.052474576 |
| Mast-Cell Activation in Asthma | 0.004703 | 0.052474576 |
| Altered Protein Expression in Cancer Metabolic Reprogramming | 0.005848 | 0.052474576 |
| Mast-Cell Activation via IgE Signaling | 0.005982 | 0.052474576 |
