# Supplementary material for: Persistent type I interferon signaling within the brain of people with HIV on ART with cognitive impairment
Source: PLoS Pathog. 2025 Aug 20;21(8):e1013411. doi: 10.1371/journal.ppat.1013411 (PMC12367146; doi:10.1371/journal.ppat.1013411)
Supplement: S12 Table — (PPTX) [file ppat.1013411.s022.pptx]

## Slide 1
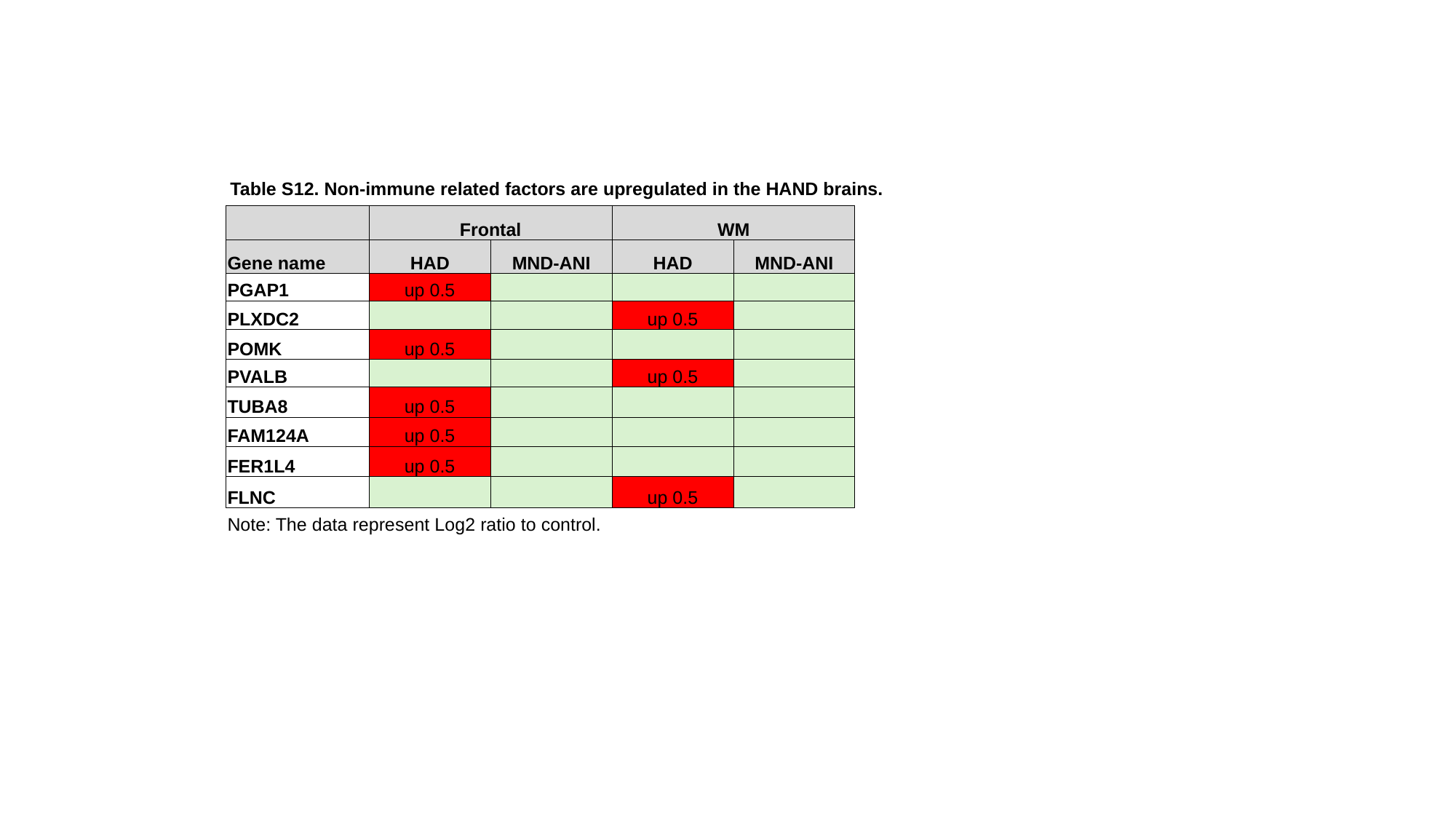

Table S12. Non-immune related factors are upregulated in the HAND brains.
| | Frontal | | WM | |
| --- | --- | --- | --- | --- |
| Gene name | HAD | MND-ANI | HAD | MND-ANI |
| PGAP1 | up 0.5 | | | |
| PLXDC2 | | | up 0.5 | |
| POMK | up 0.5 | | | |
| PVALB | | | up 0.5 | |
| TUBA8 | up 0.5 | | | |
| FAM124A | up 0.5 | | | |
| FER1L4 | up 0.5 | | | |
| FLNC | | | up 0.5 | |
Note: The data represent Log2 ratio to control.
